# Tumor-intrinsic sensitivity to the pro-apoptotic effects of IFN-γ is a major determinant of CD4^+^ CAR T-cell antitumor activity

**DOI:** 10.1038/s43018-023-00570-7

**Published:** 2023-05-29

**Authors:** Morgane Boulch, Marine Cazaux, Alexis Cuffel, Marion V. Guerin, Zacarias Garcia, Ruby Alonso, Fabrice Lemaître, Alexander Beer, Béatrice Corre, Laurie Menger, Capucine L. Grandjean, Florence Morin, Catherine Thieblemont, Sophie Caillat-Zucman, Philippe Bousso

**Affiliations:** 1 Institut Pasteur, Université de Paris Cité, INSERM U1223, Dynamics of Immune Responses Unit, Equipe Labellisée Ligue Contre le Cancer, Paris, France; 2 Université de Paris Cité, Hôpital Saint-Louis, AP-HP Nord, Laboratoire d’Immunologie, Paris, France; 3 INSERM UMR976, Institut de Recherche St-Louis, Paris, France; 4 Gustave Roussy, Villejuif, France; INSERM U1015, Villejuif, France; 5 Service d’Hémato-Oncologie, Hôpital Saint-Louis, AP-HP, Université de Paris Cité, Paris, France

**Keywords:** Imaging the immune system, Immunotherapy, Cancer

## Abstract

CD4^+^ T cells and CD4^+^ chimeric antigen receptor (CAR) T cells display highly variable antitumor activity in preclinical models and in patients; however, the mechanisms dictating how and when CD4^+^ T cells promote tumor regression are incompletely understood. With the help of functional intravital imaging, we report that interferon (IFN)-γ production but not perforin-mediated cytotoxicity was the dominant mechanism for tumor elimination by anti-CD19 CD4^+^ CAR T cells. Mechanistically, mouse or human CD4^+^ CAR T-cell-derived IFN-γ diffused extensively to act on tumor cells at distance selectively killing tumors sensitive to cytokine-induced apoptosis, including antigen-negative variants. In anti-CD19 CAR T-cell-treated patients exhibiting elevated CAR CD4:CD8 ratios, strong induction of serum IFN-γ was associated with increased survival. We propose that the sensitivity of tumor cells to the pro-apoptotic activity of IFN-γ is a major determinant of CD4^+^ CAR T-cell efficacy and may be considered to guide the use of CD4^+^ T cells during immunotherapy.

## Abstract (web summary)

Bousso and colleagues show that IFN-γ production is the dominant tumor elimination mechanism exerted by CD4^+^ CAR T cells and define tumor cell sensitivity to IFN-γ as a determinant of CD4^+^ CAR T efficacy.

## Main

While most tumor immunotherapies aimed at harnessing the killing activity of CD8^+^ T cells, there is increasing evidence that CD4^+^ T cells also play a major role during antitumor immune response both in preclinical models and in patients with cancer^1–9^.

CAR T cells, an effective therapy for B-cell malignancies and an attractive strategy under investigation for other cancers, typically uses a mixture of CD4^+^ and CD8^+^ T cells^10–14^. We and others have found that these subsets display shared and unique features^15,16^. Notably, CD4^+^ CAR T cells can exert potent antitumor activity (and even outperform CD8^+^ CAR T cells) in certain preclinical tumor models^9,17–21^. By contrast, in other settings, CD4^+^ CAR T cells show no or lower efficacy as compared to CD8^+^ CAR T cells^15,16,22–25^. Whether tumor-intrinsic parameters dictate the capacity of CD4^+^ CAR T cells to elicit tumor regression is unclear. More generally, how CD4^+^ CAR T cells eliminate tumor cells in responsive models has yet to be fully understood. Multiple mechanisms have been proposed to explain the antitumor activity of CD4^+^ T cells^2^. Although considered a hallmark of CD8^+^ T cells, CD4^+^ T cells and CD4^+^ CAR T cells can use the granzyme/perforin pathway to mediate target cell lysis^8,26–32^. This is likely facilitated in CD4^+^ CAR T cells that do not require major histocompatibility complex (MHC) class II expression on tumor cells for contact-dependent cytotoxic activity (as compared to conventional CD4^+^ T cells). Furthermore, the production of cytokines such as IFN-γ and tumor necrosis factor (TNF)-α by CD4^+^ T cells can induce tumor cell death^33^ or senescence^34^. The same cytokines could also act on the tumor vasculature to promote tumor regression^35,36^. CD4^+^ T cells have also the ability to promote macrophage tumoricidal activity^37,38^, to favor humoral responses^39^ or provide help to CD8^+^ T-cell responses^40,41^. We have recently shown that CAR T-cell-derived IFN-γ through a crosstalk with the host and the subsequent production of interleukin (IL)-12 promoted the activation of host cytotoxic effectors (CD8^+^ T cells and natural killer (NK) cells) and was also critical to sustain CD8^+^ CAR T-cell killing rate^15^; however, how these distinct mechanisms contribute to CD4^+^ CAR T-cell antitumor activity remain to be precisely quantified. More generally, we lack direct evidence of how conventional CD4^+^ T cells or CD4^+^ CAR T cells eliminate tumors.

Here, we report distinct therapeutical benefit for anti-CD19 CD4^+^ CAR T-cell therapy in two models of B-cell malignancy. Using intravital imaging of the bone marrow to quantify killing events in the sensitive tumor model, we established that perforin-dependent killing account for less than a third of tumor cell death during CD4^+^ CAR T-cell therapy. The majority of tumor cell death occurred at a distance from CAR T cells and was strictly dependent on CAR T-cell-derived IFN-γ. We provide evidence that IFN-γ acted directly on tumor cells to promote apoptosis and that tumor sensitivity to the pro-apoptotic effects of IFN-γ is a major determinant of CD4^+^ CAR T-cell efficacy.

## Results

### CD4^+^ CAR T cells exhibit tumor-dependent antitumor activity

**Fig. 1:**
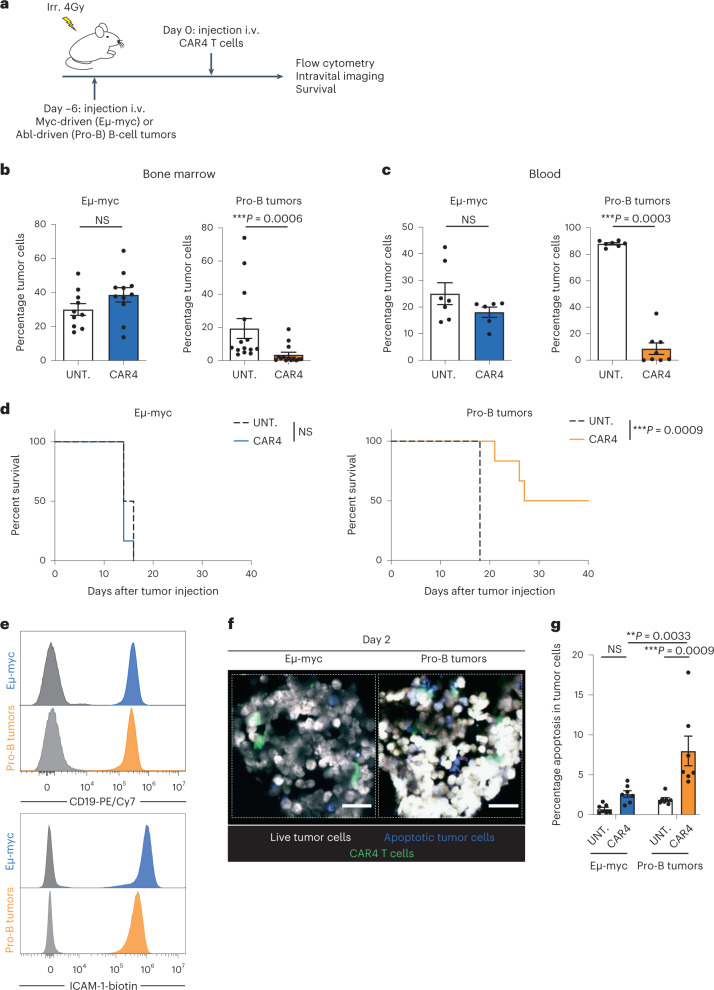
Distinct B-cell tumors exhibit differential sensitivities to CAR4 T-cell therapy. **a**, In vivo experimental setup. B-cell tumors were established by intravenous (i.v.) injection of 0.5 × 10^6^ Eμ-myc or pro-B-cell tumors expressing the FRET-based caspase 3 reporter in C57BL/6 mice after sublethal irradiation. Six days later, mice were injected intravenously with CAR4 T cells. **b**, Percentage of tumor cells recovered from the bone marrow 3 d after CAR4 T-cell transfer. Data are compiled from *n* = 3 (Eµ-myc, *n* = 10 untreated and *n* = 11 CAR4 T-cell-treated mice) or *n* = 4 (pro-B tumors, *n* = 14 untreated and *n* = 14 CAR4 T-cell-treated mice) independent experiments. Each dot represents one mouse. Two-tailed Mann–Whitney *U*-tests were used for statistical analysis. **c**, Percentage of tumor cells detected in the blood 7 d after CAR4 T-cell transfer. Representative of *n* = 2 independent experiments (for Eµ-myc, *n* = 7 untreated and *n* = 6 CAR4 T-cell-treated mice; for pro-B tumors, *n* = 7 untreated and *n* = 8 CAR4 T-cell-treated mice). Each dot represents one mouse. Two-tailed Mann–Whitney *U*-tests were used for statistical analysis. **d**, CAR4 T-cell therapy prolonged the survival of pro-B tumor- but not Eµ-myc tumor-bearing mice. Log-rank test was used for statistical analysis (*n* = 6 mice per group). Representative of *n* = 2 (Eµ-myc) or *n* = 4 (pro-B tumors) independent experiments. **e**, CD19 and ICAM-1 (CD54) expression on Eµ-myc and pro-B-cell tumors. Gray histograms represent the unstained control. **f**, Representative two-photon images of the bone marrow of tumor-bearing mice treated with CAR4 T cells 2 d earlier. CAR4 T cells are shown in green, live tumor cells in white, and apoptotic tumor cells in blue. Scale bars, 30 µm. Representative of *n* = 2 independent experiments. **g**, Bone marrow composition was analyzed ex vivo by flow cytometry 3 d after CAR4 T-cell transfer. Summary graphs showing the percentage of apoptotic tumor cells. Data are compiled from *n* = 2 independent experiments (*n* = 7 mice per group). Each dot represents one mouse. Two-way analysis of variance (ANOVA) and Tukey’s multiple comparisons were used for statistical analysis. Data are expressed as mean ± s.e.m. ***P < 0.001; **P < 0.01; NS, not significant. Source data

**Extended Data Fig. 1:**
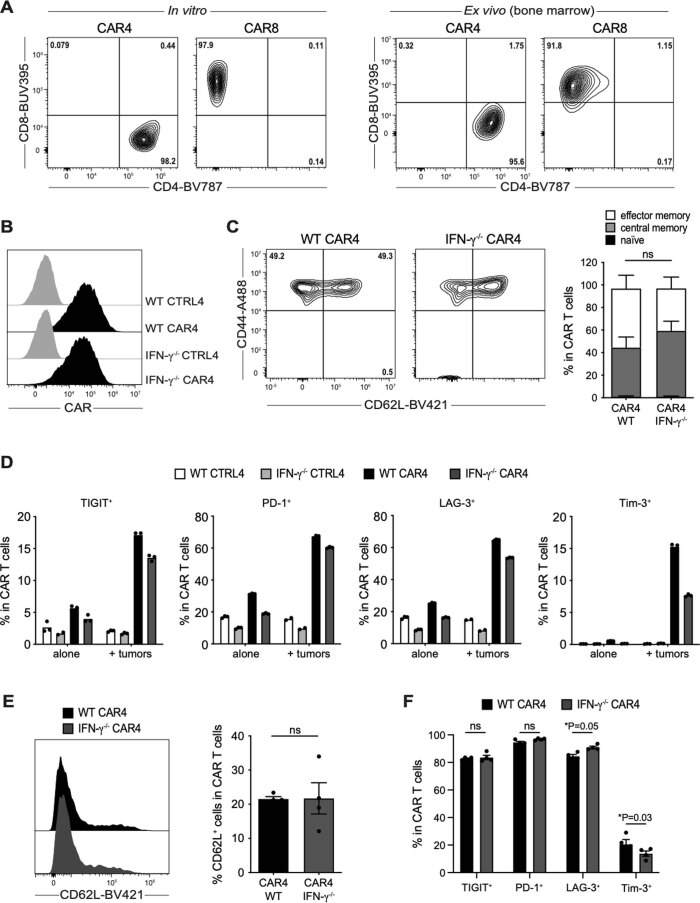
*In vitro* and *ex vivo* phenotyping of CAR T cells. (**a**) CD4 and CD8 surface expression by CAR4 and CAR8 T cells (representative of n = 6 independent experiments). (**b**) CAR expression by WT and IFN-γ^−/−^ CD4^+^ T cells (representative of n = 8 independent experiments). CTRL4, untransduced control CD4^+^ T cells. (**c**) Representative FACS plot (left) and bar graphs (right) showing the percentage of effector/effector memory (CD44^+^ CD62L^−^), central memory (CD44^+^ CD62L^+^) and naive T cells (CD44^−^ CD62L^+^) in WT and IFN-γ^−/−^ CAR4 T cells (pooled from n = 3 independent experiments). Two-way ANOVA and Sidak’s multiple comparisons were used for statistical analysis. (**d**) Percentage of TIGIT, PD-1, LAG-3 and Tim-3 positive cells in WT and IFN-γ^−/−^ CAR4 T cells and their untransduced counterparts (CTRL4) after co-culture (or not) with pro-B-DEVD tumors at 1:1 effector-to-target ratio for 24 hours. Each dot represents one technical replicate (n = 1 experiment). (**e, f**) *Ex vivo* analysis of CAR4 T cells recovered from the bone marrow of pro-B-cell tumor-bearing mice 3 days after the treatment with WT or IFN-γ^−/−^ CAR4 T cells (10 × 10^6^). (**e**) Representative histograms (left) and bar graphs (right) showing the percentage of CD62L expressing cells in WT and IFN-γ^−/−^ CAR4 T cells. Each dot represents one mouse (n = 4 mice per group). Two-tailed Mann-Whitney U-test was used for statistical analysis. (**f**) Percentage of TIGIT, PD-1, LAG-3 and Tim-3 positive cells in WT and IFN-γ^−/−^ CAR4 T cells recovered from the bone marrow 3 days after their transfer. Each dot represents one mouse (n = 4 mice per group). Two-way ANOVA and Sidak’s multiple comparisons were used for statistical analysis. Data are expressed as mean ± SEM. *P<0.05; ns, not significant. Source data

**Extended Data Fig. 2:**
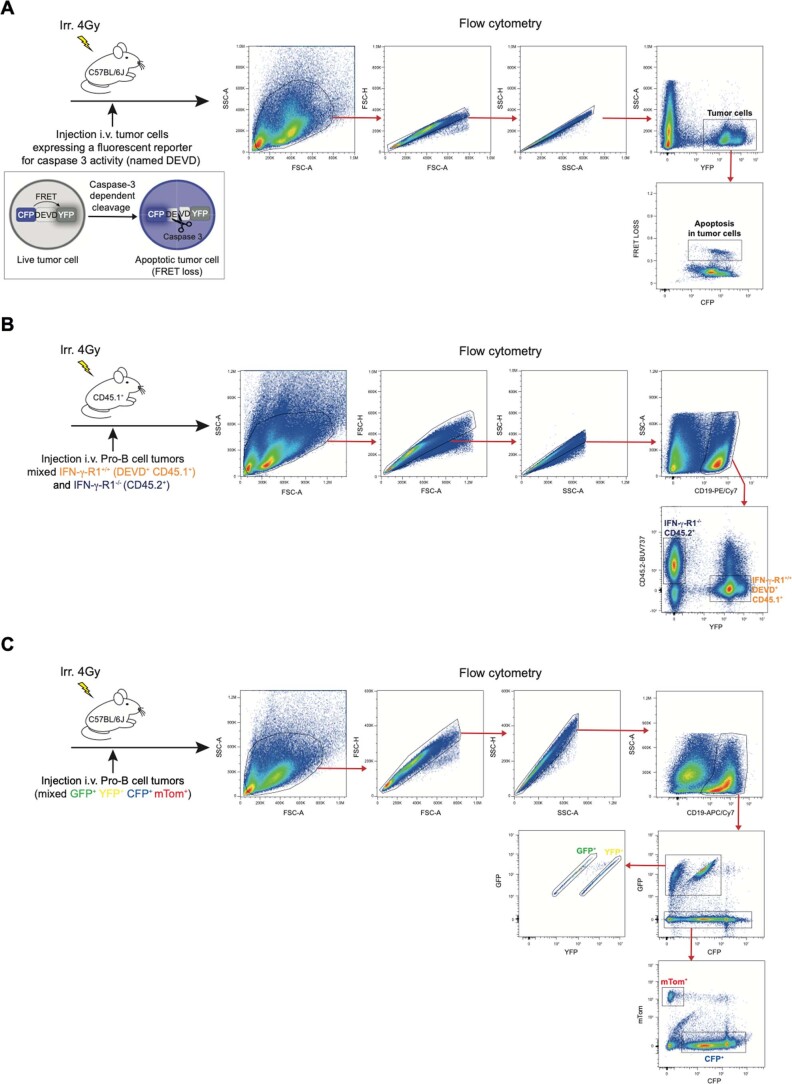
Gating strategies for flow cytometry identification of pro-B-cell tumors. (**a**) Gating strategy for identifying pro-B-cell tumors expressing the FRET-based caspase 3 reporter (named DEVD) and tumor apoptosis in the bone marrow of tumor-bearing mice. CFP and YFP are linked by the target peptide DEVD, which is cleaved upon caspase 3 activation, resulting in loss of FRET in apoptotic cells. (**b**) Gating strategy for identifying CD45.1^+^ IFN-γ-R1^+/+^ and CD45.2^+^ IFN-γ-R1^−/−^ pro-B-cell tumors in the bone marrow of tumor-bearing mice. (**c**) Gating strategy for identifying CFP^+^, GFP^+^, YFP^+^ and mTom^+^ pro-B-cell tumors in the bone marrow of tumor-bearing mice.

The ability of CD4^+^ CAR T cells (CAR4 T cells) to induce tumor regression has been shown to be highly variable in distinct preclinical models of B-cell malignancies or solid tumors. In particular, we have previously shown that CD4^+^ CAR T cells are poorly effective in a model of Myc-driven B-cell lymphoma (Eμ-myc), whereas CD8^+^ CAR T cells (CAR8 T cells) readily induced tumor regression^15,42^. To test an additional model of B-cell malignancy, we used Abelson-driven pro-B-cell tumors^43^ (a model of B-cell acute lymphoblastic leukemia) in the same settings of anti-CD19 CAR4 T-cell therapy in vivo (Fig. 1a and Extended Data Fig. 1a). In mice with established pro-B-cell tumors, CAR4 T cells substantially reduced tumor burden in the bone marrow and blood (Fig. 1b,c and Extended Data Fig. 2a). As expected, CAR4 T cells were incapable of controlling tumor growth in mice bearing Eμ-myc tumors. Consistently, CAR4 T cells increased mouse survival in the pro-B-cell model but did not confer any survival advantage in the Eμ-myc model (Fig. 1d). Of note, levels of CD19 expression or of the adhesion molecule ICAM-1 (typically involved in effector–target contact stabilization) could not account for the differential antitumor responses seen in these two models (Fig. 1e) as values were similar at steady state. Next, we thought to assess whether CAR4 T-cell-mediated tumor control was associated with a higher frequency of tumor cell death. To this end, we relied on intravital imaging of the bone marrow and flow cytometry using Eμ-myc or pro-B-cell tumors expressing a FRET-based caspase 3 reporter. Caspase 3 activity was detected in CAR4 T-cell-treated mice harboring pro-B-cell tumors but was minimal in treated mice with Eμ-myc tumors or in untreated animals (Fig. 1f,g). Together, these experiments prompted us to explore in more detail the mechanisms dictating the antitumor activity of CAR4 T cells against B-cell tumors.

### CAR4 T cells rely on IFN-γ to control pro-B-cell tumors

**Fig. 2:**
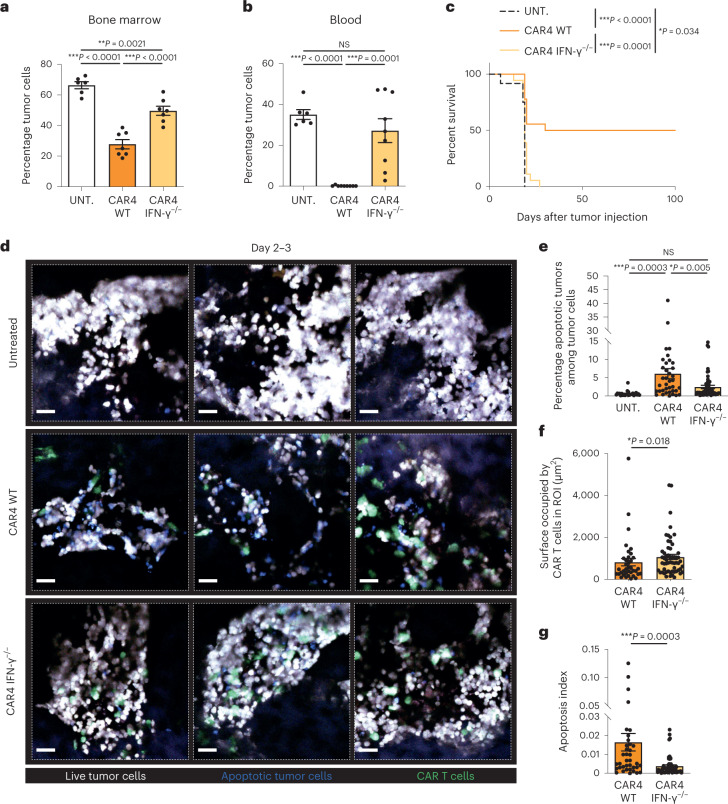
CAR4 T cells rely on IFN-γ to control pro-B-cell tumors. Pro-B-cell tumors were established by intravenous injection of 0.5 × 10^6^ Pro-B-DEVD cells in C57BL/6 mice after sublethal irradiation. Six days later, mice were injected intravenously with WT or IFN-γ^−/−^ CAR4 T cells or left untreated. **a**, Percentage of tumor cells recovered from the bone marrow 3 d after the transfer of WT or IFN-γ^−/−^ CAR4 T cells. Each dot represents one mouse (*n* = 6 untreated, *n* = 7 WT CAR4 T-cell-treated and *n* = 7 IFN-γ^−/−^ CAR4 T-cell-treated mice from two independent experiments). One-way ANOVA was used for statistical analysis. **b**, Percentage of tumor cells detected in the blood 7 d after the transfer of WT or IFN-γ^−/−^ CAR4 T cells. Each dot represents one mouse (*n* = 6 untreated, *n* = 9 WT CAR4 T-cell-treated and *n* = 9 IFN-γ^−/−^ CAR4 T-cell-treated mice). One-way ANOVA was used for statistical analysis. **c**, WT CAR4 but not IFN-γ^−/−^ CAR4 T-cell therapy prolonged mouse survival. Log-rank test was used for statistical analysis. Data are compiled from two independent experiments (*n* = 12 untreated, *n* = 18 WT CAR4 T-cell-treated and *n* = 18 IFN-γ^−/−^ CAR4 T-cell-treated mice). **d**–**g**, Killing activity of WT or IFN-γ-deficient CAR4 T cells was assessed by intravital imaging of the bone marrow on days 2 and 3 after CAR T-cell transfer. Representative two-photon images of the bone marrow of pro-B tumor-bearing mice treated with WT or IFN-γ^−/−^ CAR4 T cells (**d**). CAR T cells are shown in green, live tumor cells in white and apoptotic tumor cells in blue. Scale bars, 30 µm. Quantification of the percentage of apoptotic tumors (ratio of the surface occupied by apoptotic tumors to the total surface occupied by tumor cells) (**e**) and the surface occupied by CAR T cells in the ROI (**f**). An apoptosis index was calculated for WT and IFN-γ^−/−^ CAR4 T cells by normalizing the percentage of apoptotic tumors as calculated in **e** to the surface occupied by CAR T cells as calculated in **f** (**g**). Data shown in **d**–**g** are compiled from *n* = 2 independent experiments. Each dot represents an individual tumor region (*n* = 25 tumor regions from *n* = 3 untreated mice, *n* = 37 tumor regions from *n* = 3 WT CAR4 T-cell-treated mice and *n* = 57 tumor regions from *n* = 4 IFN-γ^−/−^ CAR4 T-cell-treated mice). One-way ANOVA (**e**) and two-tailed Mann–Whitney *U*-tests (**f**,**g**) were used for statistical analysis. Data are expressed as mean ± s.e.m. ***P < 0.001; **P < 0.01; *P < 0.05; NS, not significant. Source data

Given the importance of IFN-γ in antitumor responses, we asked whether the capacity of CAR4 T cells to produce IFN-γ was essential for the control of pro-B-cell tumors. Mice with established tumors were therefore treated with CAR4 T cells that were either competent or deficient for IFN-γ. As shown in Fig. 2a,b, the capacity of CAR4 T cells to control tumor burden in the bone marrow and in the blood was largely reduced when IFN-γ^−/−^ CAR4 T cells were used for treatment. In addition, while wild-type (WT) CAR4 T cells promoted durable tumor control in approximately half of the animals, IFN-γ^−/−^ CAR4 T cells provided little if any survival benefit (Fig. 2c). Of note, WT and IFN-γ^−/−^ CAR4 T cells expressed similar levels of CAR, activation and exhaustion markers at their surface ex vivo (Extended Data Fig. 1b–f). Using intravital imaging, we observed that IFN-γ^−/−^ CAR4 T cells induced lower levels of caspase 3 activity in tumor cells compared to WT CAR4 T-cell-treated mice (Fig. 2d–g). These differences were not accounted for by a distinct accumulation at the tumor site as IFN-γ^−/−^ CAR4 T cells accumulated to a slightly higher degree than WT CAR4 T cells (Fig. 2f). Of note, apoptosis events were largely due to the presence of CAR4 T cells as caspase 3 activity was barely detectable in untreated animals (Fig. 2d,e). In sum, our results established that CAR4 T-cell-derived IFN-γ was important for inducing tumor cell death and promoting control of pro-B-cell tumors.

### Elimination of tumors by CAR4 T cells without cell contact

**Fig. 3:**
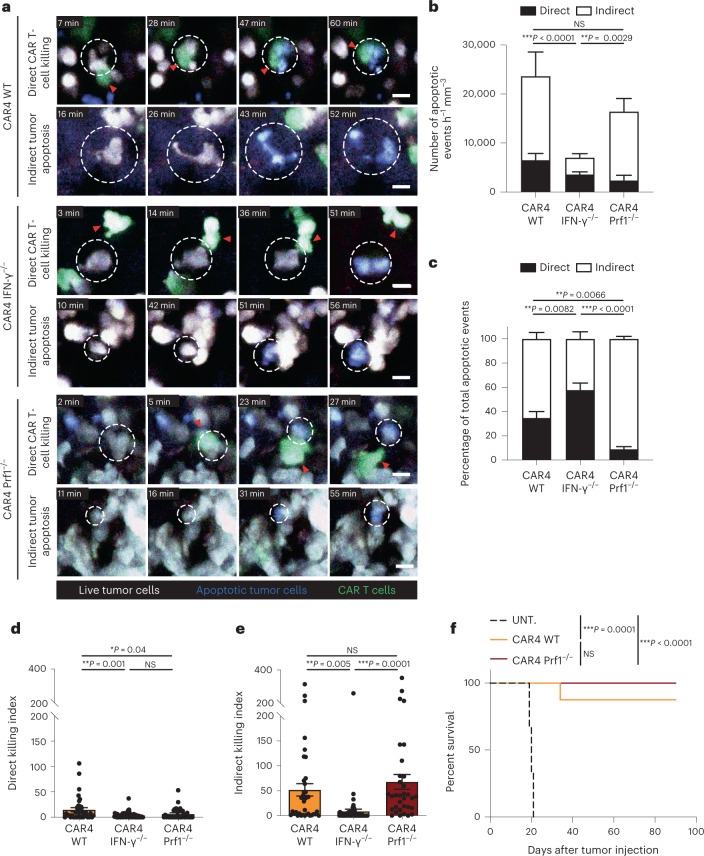
CAR4 T cells eliminate B-cell tumors using both IFN-γ-dependent and perforin-dependent mechanisms. Pro-B-cell tumors were established in C57BL/6 mice after sublethal irradiation. Six days later, mice were injected with WT, IFN-γ^−/−^ or Prf1^−/−^ CAR4 T cells. **a**, Two-photon timelapse images showing direct and indirect tumor apoptotic events mediated by CAR4 T cells. Apoptotic events (detected by the FRET-based reporter for caspase 3 activity) were classified as direct killing when a CAR T cell contacted the target cell before FRET loss detection. Indirect events corresponded to tumor cells showing FRET loss without detectable interactions with CAR T cells. Red arrowheads show CAR T cells associated with killing. White dashed circles illustrate tumor cells undergoing apoptosis. CAR T cells are shown in green, live tumor cells in white, and apoptotic tumor cells in blue. Scale bars, 10 µm. **b**,**c**, Quantification (normalized per hour and per surface area) (**b**) and proportion (**c**) of tumor apoptotic events. A total of *n* = 216, *n* = 126 and *n* = 259 apoptotic events were recorded for WT, IFN-γ^−/−^ and Prf1^−/−^ CAR4 T cells, respectively. Two-way ANOVA and Sidak’s multiple comparisons were used for statistical analysis. **d**, A direct killing index was calculated as the ratio of normalized direct apoptotic events to the surface occupied by CAR T cells in each image. Each dot represents one tumor region (*n* = 37, *n* = 57, *n* = 33 for WT, IFN-γ^−/−^ and Prf1^−/−^ CAR4 T cells, respectively). One-way ANOVA and Tukey’s multiple comparisons were used for statistical analysis. **e**, An indirect killing index was calculated as the ratio of normalized indirect apoptotic events to the surface occupied by CAR T cells in each image. Each dot represents one tumor region. One-way ANOVA and Tukey’s multiple comparisons were used for statistical analysis. Compiled from multiple regions imaged after the transfer of WT CAR4 T cells (*n* = 3 mice, *n* = 26 h of video analyzed), IFN-γ^−/−^ CAR4 T cells (*n* = 4 mice, *n* = 42 h of video analyzed) and Prf1^−/−^ CAR4 T cells (*n* = 3 mice, *n* = 46 h of video analyzed) from *n* = 2 independent experiments. **f**, WT and Prf1^−/−^ CAR4 T cells similarly prolong mouse survival. Log-rank test was used for statistical analysis (*n* = 6 untreated, *n* = 8 WT CAR4 T-cell-treated and *n* = 9 Prf1^−/−^ CAR4 T-cell-treated mice). Data are expressed as mean ± s.e.m. ***P < 0.001; **P < 0.01; *P < 0.05; NS, not significant. Source data

**Extended Data Fig. 3:**
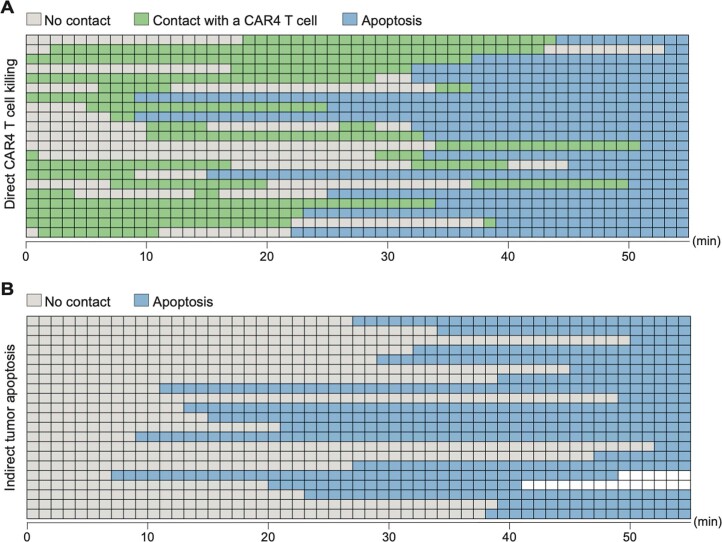
Kinetics of tumor apoptotic events mediated by CAR4 T cells. Pro-B-cell tumors were established by intravenous injection of 0.5 × 10^6^ pro-B-DEVD tumor cells in C57BL/6 mice after sublethal irradiation. Six days later, mice were injected intravenously with WT CAR4 T cells and subjected to intravital two-photon imaging of the bone marrow. Apoptotic events (detected by the genetically-encoded FRET-based reporter for caspase 3 activity) were classified as direct killing when a CAR T cell engaged the target cell before FRET loss detection. Indirect events corresponded to tumor cells undergoing FRET loss without any apparent interactions with a CAR T cell. (**a**) Kinetics of direct CAR4 T cell-mediated killing events. (**b**) Kinetics of indirect tumor apoptotic events. Each line (n = 21) represents an individual tumor cell undergoing apoptosis during the imaging period. Gray squares represent periods without contact, green squares represent periods during which tumor cells are contacting a CAR4 T cell and blue squares represent periods during which tumor cells appear apoptotic. Data are representative of n = 26 hours of movie analyzed, from n = 3 mice from n = 3 independent experiments.

**Extended Data Fig. 4:**
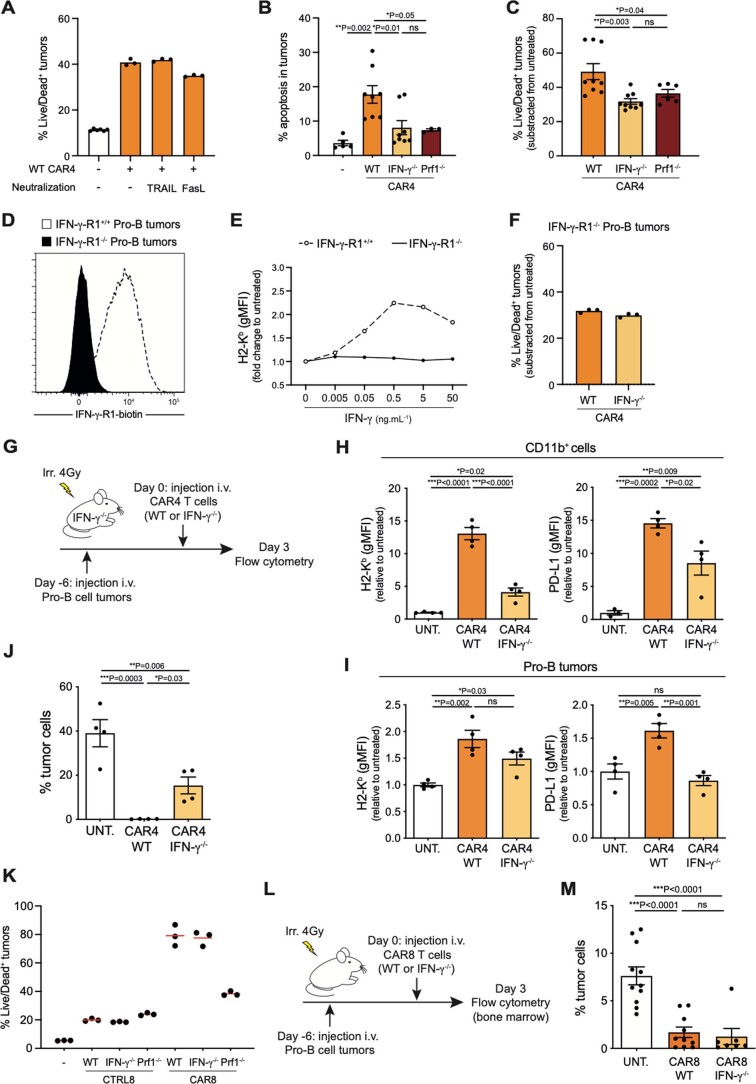
Role of IFN-γ production for the activity of CAR4 and CAR8 T cells. (**a-f**) *In vitro* quantification of CAR4 T cell cytotoxic activity. (**a**) Pro-B-DEVD tumors were co-cultured with WT CAR4 T cells at 1:1 effector-to-target ratio or left untreated for 24 hours. When indicated, TRAIL and FasL were neutralized using a blocking antibody. Tumor cell death was assessed using fixable viability dye (Zombie NIR). Each dot represents one technical replicate. Data are representative of n = 2 independent experiments. (**b, c**) Pro-B-DEVD tumors were co-cultured with WT, IFN-γ^−/−^ or Prf1^−/−^ CAR4 T cells at 1:1 effector-to-target ratio or left untreated for 24 hours. (**b**) Tumor cell apoptosis was assessed using the FRET-based caspase 3 reporter. (**c**) Tumor cell death was assessed using fixable viability dye (Zombie NIR) and normalized to untreated tumor cells. (**b–c**) Each dot represents one technical replicate from n = 2 independent experiments for Prf1^−/−^ CAR4 T cells and n = 3 independent experiments for WT and IFN-γ^−/−^ CAR4 T cells. One-way ANOVA and Holm-Sidak’s multiple comparisons were used for statistical analysis. (**d**) Representative histograms of IFN-γ-R1 expression by WT (white) and CRISPR/Cas9 edited pro-B-cell tumors (black). (**e**) Functional validation of CRISPR/Cas9-mediated deletion of IFN-γ-R1 on pro-B-cell tumors. Pro-B-cell tumors were incubated with the indicated IFN-γ concentrations *in vitro* for 24 hours. H2-K^b^ surface expression analyzed by flow cytometry and expressed as a fold change relative to untreated cells. Each dot represents the mean of 3 technical replicates (n = 1 experiment). gMFI, geometric mean fluorescence intensity. (**f**) IFN-γ-R1^−/−^ pro-B-cell tumors were co-cultured with WT or IFN-γ^−/−^ CAR4 T cells at 1:1 effector-to-target ratio. Tumor cell death was assessed using fixable viability dye (Zombie NIR) and normalized to untreated tumor cells. Each dot represents one technical replicate (n = 1 experiment). (**g**) *In vivo* experimental setup. Pro-B-cell tumors were established by intravenous injection of 0.5 × 10^6^ of pro-B cells expressing the FRET-based caspase 3 reporter in IFN-γ^−/−^ mice after sublethal irradiation. Six days later, mice were injected intravenously with WT or IFN-γ^−/−^ CAR4 T cells. Three days after CAR T cell transfer, bone marrow cells were processed using flow cytometry. (**h, i**) CAR4 T cell-derived IFN-γ increases H2-K^b^ and PD-L1 levels in tumor and tumor-infiltrating immune cells. Surface expression of H2-K^b^ (left) and PD-L1 (right) was assessed on CD11b^+^ myeloid cells (**H**) and tumor cells (**I**). gMFI, geometric mean fluorescence intensity. Each dot represents one mouse (n = 4 mice per group from n = 1 experiment). One-way ANOVA, Tukey’s and Holm-Sidak’s multiple comparisons were used for statistical analysis. (**j**) Percentage of tumor cells recovered from the bone marrow. Each dot represents one mouse (n = 4 mice per group from n = 1 experiment). One-way ANOVA and Holm-Sidak’s multiple comparisons were used for statistical analysis. (**k**) *In vitro* quantification of CAR8 T cell cytotoxic activity. Pro-B-DEVD tumors were co-cultured with WT, IFN-γ^−/−^ or Prf1^−/−^ CAR8 T cells or untransduced control CD8^+^ T cells (CTRL8) at 1:1 effector-to-target ratio for 24 hours. Tumor cell death was assessed using fixable viability dye (Zombie NIR). Each dot represents one technical replicate (n = 1 experiment). (**l**) Pro-B-cell tumors were established by intravenous injection of 0.5 × 10^6^ pro-B-DEVD cells in C57BL/6 mice after sublethal irradiation. Six days later, mice were injected intravenously with WT or IFN-γ^−/−^ CAR8 T cells. (**m**) Percentage of tumor cells recovered from the bone marrow 3 days after the transfer of CAR8 T cells. Each dot represents one mouse (n = 11 untreated and n = 10 WT CAR8 T cell-treated mice from n = 3 independent experiments, n = 7 IFN-γ^−/−^ CAR8 T cell-treated mice from n = 2 independent experiments). One-way ANOVA and Tukey’s multiple comparisons were used for statistical analysis. Data are expressed as mean ± SEM. ***P<0.001; **P<0.01; *P<0.05; ns, not significant. Source data

To gain additional insight into how CAR4 T cells contribute to tumor destruction, we performed intravital imaging of the bone marrow in mice with established pro-B-cell tumors and treated with WT or IFN-γ^−/−^ or perforin-deficient (Prf1^−/−^) CAR4 T cells. We used the caspase 3 reporter to identify the occurrence of tumor apoptosis during the imaging period. Tumor killing events were classified as ‘direct’ when happening in the context of a contact with a CAR4 T cell and ‘indirect’ when tumor cell death occurred in the absence of detectable CAR4 T-cell contact (Fig. 3a, Extended Data Fig. 3 and Supplementary Video 1). In mice treated with WT CAR4 T cells, a third of tumor apoptosis events were associated with a CAR4 T-cell contact (Fig. 3b–c and Supplementary Video 2), suggesting that CAR4 T cells can exhibit some degree of direct cytotoxicity; however, the majority of tumor cell death occurred at distance from CAR4 T cells (Fig. 3b,c and Supplementary Video 2), suggesting a distinct mechanism for tumor elimination. Notably, when using IFN-γ^−/−^ CAR4 T cells for treatment, most of the indirect killing events were lost (Fig. 3b,c and Supplementary Video 3). Quantitatively, CAR4 T-cell-derived IFN-γ strongly promoted indirect killing on a per CAR4 T-cell basis (Fig. 3d,e). Of note, there was a reduction in direct killing when using IFN-γ^−/−^ CAR4 T cells, possibly due to the importance of the IFN-γ–IL-12 axis for maintaining CAR T-cell cytotoxic potential^15^. Conversely, we assessed the killing mode of Prf1^−/−^ CAR4 T cells. In these settings, indirect killing events were detected at similar rate as compared to WT CAR4 T cells (Fig. 3b; NS, *P* = 0.9312) but direct killing events were largely abrogated (Fig. 3b,e and Supplementary Video 4). This observation suggested in particular that killing scored as ‘indirect’ were unlikely contact-dependent killing in which the lethal hit was not visualized. Thus, direct but not indirect killing events were dependent on the perforin/granzyme activity of CAR4 T cells. The lower contribution of the perforin pathway (over the IFN-γ pathway) to tumor killing by CAR4 T cells was also reflected by the fact Prf1^−/−^ CAR4 T cells displayed similar therapeutic benefit as compared to WT CAR4 T cells (Fig. 3f). The residual direct killing activity seen when using Prf1^−/−^ CAR4 T cells may possibly be due to Fas-dependent killing (Extended Data Fig. 4a). We also assessed the occurrence of IFN-γ-dependent and Prf1-dependent killing by CAR4 T cells in vitro. As shown in Extended Data Fig. 4b,c, tumor killing was reduced when using IFN-γ^−/−^ or Prf1^−/−^ CAR4 T cells, confirming the involvement of both pathways. Of note, the contribution of Prf1-dependent killing tended to be higher in vitro compared to in vivo, possibly because the assay favored close contacts. Consistently, CRISPR/Cas9-mediated removal of IFN-γ-R1 on pro-B-cell tumors also reduced overall killing by CAR4 T cells (Extended Data Fig. 4d–f). In sum, our in vivo results suggest that CAR4 T cells mostly eliminate tumors remotely in an IFN-γ-dependent mechanism and to a lesser extent during direct tumor cell contact in a perforin-dependent manner.

### CAR4 T-cell-derived IFN-γ is sensed by immune and tumor cells

**Fig. 4:**
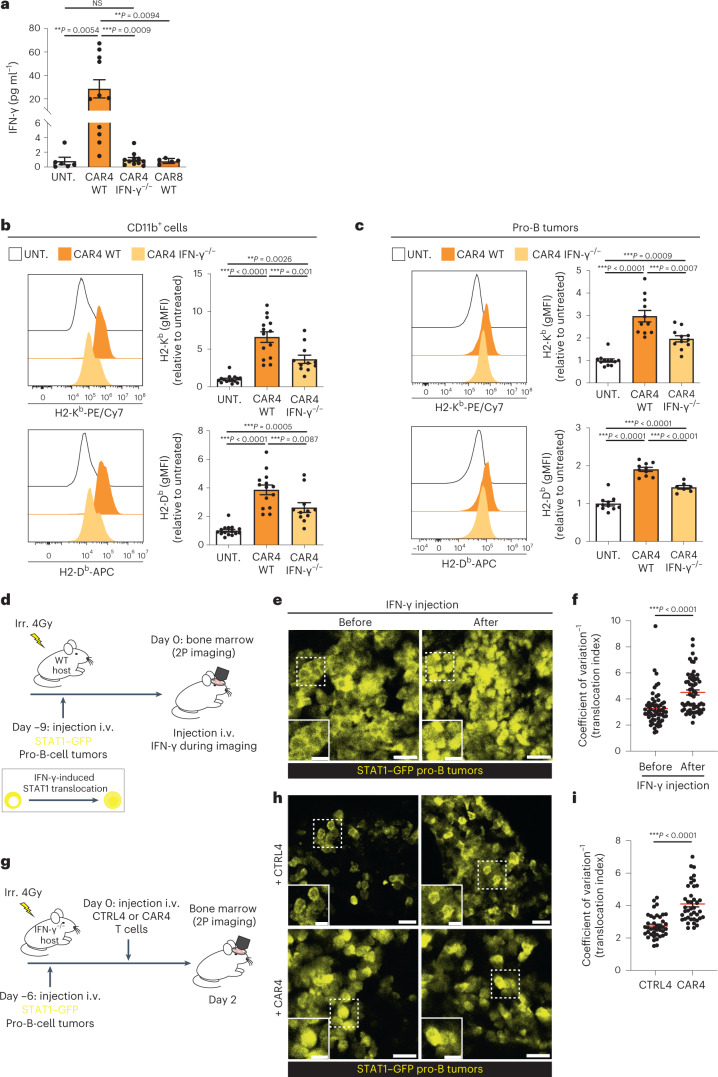
CAR4 T-cell-derived IFN-γ diffuses extensively in the tumor microenvironment. **a**–**c**, Pro-B-cell tumors were established in C57BL/6 mice after sublethal irradiation. Six days later, mice were injected with the indicated population of CAR T cells. CAR4 T cells represent the major source of IFN-γ (**a**). Serum IFN-γ was measured 3 d after CAR T-cell transfer. Each dot represents one mouse (*n* = 6 untreated, *n* = 11 WT CAR4 T-cell-treated and *n* = 11 IFN-γ^−/−^ CAR4 T-cell-treated mice from *n* = 3 independent experiments and *n* = 5 WT CAR8 T-cell-treated mice from *n* = 2 independent experiments). One-way ANOVA and Tukey’s multiple comparisons were used for statistical analysis. MHC class I molecule upregulation was assessed on CD11b^+^ myeloid cells (**b**) and tumor cells (**c**) 3 d after cell transfer. Pooled from *n* = 4 (*n* = 15 untreated, *n* = 14 WT CAR4 T-cell-treated and *n* = 11 IFN-γ^−/−^ CAR4 T-cell-treated mice) (**b**) and *n* = 3 (*n* = 11 untreated, *n* = 10 WT CAR4 T-cell-treated and *n* = 7 IFN-γ^−/−^ CAR4 T-cell-treated mice) (**c**) independent experiments. Each dot represents one mouse. One-way ANOVA and Tukey’s multiple comparisons were used for statistical analysis. **d**, Tumors were established by injection of pro-B cells expressing a STAT1–GFP reporter into C57BL/6 mice after sublethal irradiation. Nine days later, mice were subjected to intravital imaging of the bone marrow before and after the i.v. injection of IFN-γ (10 µg). 2P, two-photon. **e**, Two-photon images showing the nuclear translocation of STAT1 after the injection of IFN-γ. Scale bars, 20 µm. **f**, A translocation score was computed for tumor cells before or after IFN-γ injection. Each dot represents one cell (*n* = 60 cells). Two-tailed Mann–Whitney *U*-test was used for statistical analysis. **g**, Pro-B-cell tumors were established by injection of pro-B cells expressing STAT1–GFP reporter into IFN-γ^−/−^ mice after sublethal irradiation. Mice were injected with CAR4 T cells or untransduced CD4^+^ T cells (CTRL4) and subjected to intravital imaging of the bone marrow 2 d later. **h**, Two-photon images showing the nuclear translocation of STAT1 in tumor cells upon the treatment with CAR4 T cells but not CTRL4 T cells. Scale bars, 20 µm. **i**, The translocation score was computed from mice treated with CTRL4 (*n* = 40 cells, representative of *n* = 5 videos) or CAR4 (*n* = 40 cells, representative of *n* = 10 videos) T cells. Each dot represents one cell. Two-tailed Mann–Whitney *U*-test was used for statistical analysis. Data are expressed as mean ± s.e.m. ***P < 0.001; **P < 0.01; *P < 0.05; NS, not significant. Source data

Having shown the importance of IFN-γ for tumor regression, we confirmed the presence of IFN-γ during CAR4 T-cell therapy. To this end, we measured systemic IFN-γ concentration in the serum of mice treated with WT or IFN-γ^−/−^ CAR4 T cells. As shown in Fig. 4a, IFN-γ concentration substantially increased upon injection of WT CAR4 T cells but not upon treatment with IFN-γ^−/−^ CAR4 T cells, indicating that CAR4 T cells represent the major source of IFN-γ. Consistently, WT CAR4 T cells but not IFN-γ^−/−^ CAR4 T cells were perfectly able to control pro-B-cell tumors in IFN-γ^−/−^ hosts, suggesting that host-derived IFN-γ does not play a major role in CAR4 T-cell therapeutic activity (Extended Data Fig. 4g–j).

By comparison, IFN-γ concentrations measured upon CAR8 T-cell treatment were >30 fold lower, highlighting the role of CD4^+^ CAR T cells in generating an IFN-γ-rich milieu (Fig. 4a). This observation is also consistent with the fact the CAR8 T cells were able to control pro-B-cell tumors independently of IFN-γ in vitro and in vivo, and relied primarily on the perforin pathway for killing (Extended Data Fig. 4k–m).

**Extended Data Fig. 5:**
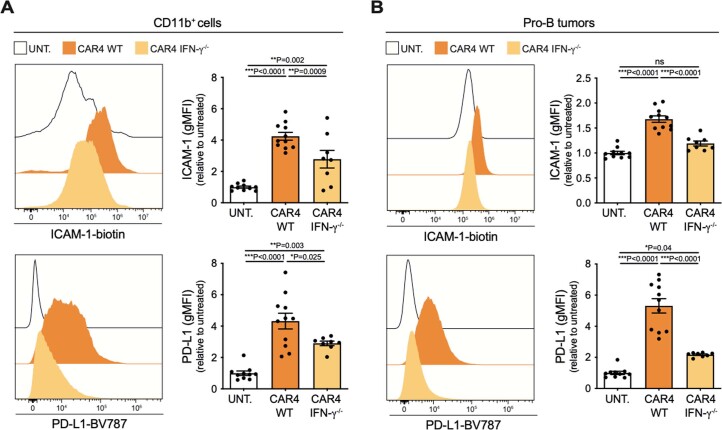
CAR4 T cells induce upregulation of ICAM-1 and PD-L1 molecules on both immune and tumor cells in the tumor microenvironment. Pro-B-cell tumors were established by intravenous injection of 0.5 × 10^6^ pro-B-DEVD cells in C57BL/6 mice after sublethal irradiation. Six days later, mice were injected intravenously with WT or IFN-γ^−/−^ CAR4 T cells (5–10 × 10^6^). (**a–b**) CAR4 T cell-derived IFN-γ induces ICAM-1 and PD-L1 upregulation in both tumor and tumor-infiltrating immune cells. Bone marrow cells were analyzed using flow cytometry 3 days after CAR T cell transfer. Surface expression of ICAM-1 and PD-L1 was assessed on CD11b^+^ myeloid (**A**) and tumor cells (**B**). gMFI, geometric mean fluorescence intensity. Each dot represents one mouse (n = 10 untreated and n = 11 WT CAR4 T cell-treated mice from n = 3 independent experiments, n = 8 IFN-γ^−/−^ CAR4 T cell-treated mice from n = 2 independent experiments). One-way ANOVA and Tukey’s multiple comparisons were used for statistical analysis. Data are expressed as mean ± SEM. ***P<0.001; **P<0.01; *P<0.05; ns, not significant. Source data

To assess the impact of IFN-γ production at the tumor site, we analyzed MHC class I, ICAM-1 and PD-L1 expression, which are classically upregulated by IFNs. CAR4 T-cell therapy resulted in upregulation of all these markers in both tumor and immune cells and this was partly due to CAR4 T-cell-derived IFN-γ (Fig. 4b,c and Extended Data Fig. 5), possibly due to the additional contribution of type I IFNs. To directly test the extent of IFN-γ diffusion in the tumor microenvironment, we introduced a fluorescent reporter for STAT1 activity^44^ in pro-B-cell tumors (Fig. 4d). Using intravital imaging of the bone marrow, we confirmed that injection of recombinant IFN-γ in mice with established tumors resulted in STAT1 translocation in most tumor cells (Fig. 4e,f). We then assessed STAT1 activity in tumor-bearing mice treated with CAR4 T cells or control CD4^+^ T cells (CTRL4) (Fig. 4g). We used IFN-γ^−/−^ hosts in these experiments to specifically evaluate the diffusion of CAR T-cell-derived IFN-γ. As shown in Fig. 4h,i, transfer of CAR4 but not CTRL4 T cells resulted in STAT1 translocation throughout the tumor. These observations indicate that IFN-γ acts broadly in the tumor microenvironment, most likely due to the efficient diffusion of this cytokine^44,45^.

### CAR4 T-cell-derived IFN-γ promotes tumor cell apoptosis

**Fig. 5:**
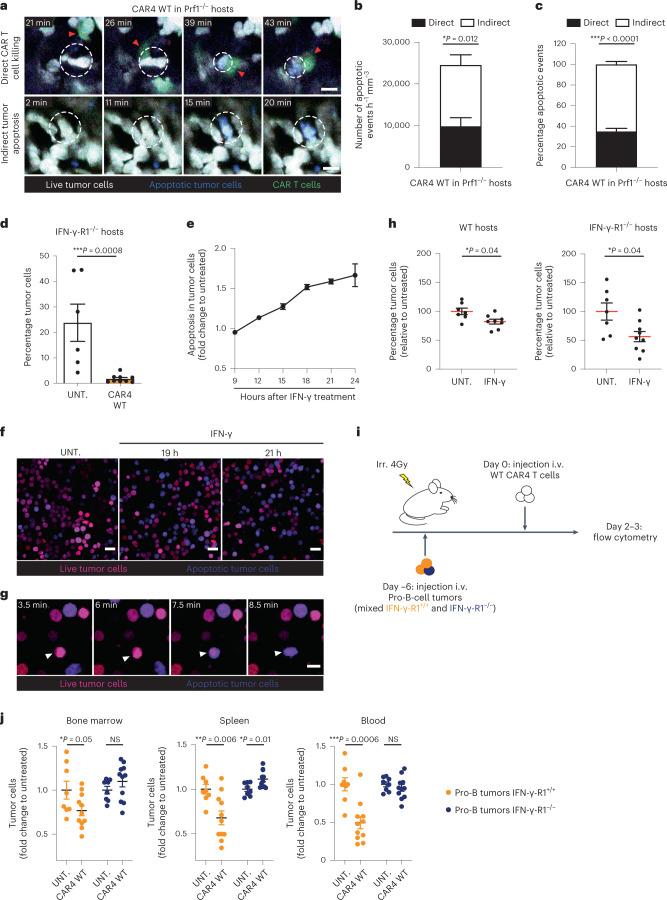
CAR4 T-cell-derived IFN-γ directly acts on tumor cells to promote tumor apoptosis. **a**–**c**, Pro-B-cell tumors were established in Prf1^−/−^ mice and treated with CAR4 T cells. Two-photon timelapse images illustrating direct and indirect tumor apoptotic events (**a**). Scale bars, 10 µm. Quantification (normalized per hour per imaged surface) (**b**) and proportion (**c**) of direct and indirect tumor apoptotic events (*n* = 540 apoptotic events, *n* = 4 mice, *n* = 51.8 h of video analyzed) from *n* = 3 independent experiments. Two-tailed Mann–Whitney *U*-tests were used for statistical analysis. **d**, Tumor-bearing IFN-γ-R1^−/−^ hosts were treated with CAR4 T cells or left untreated. Percentage of tumor cells recovered from the bone marrow 3 d after cell transfer. Data were compiled from *n* = 2 independent experiments (*n* = 6 untreated and *n* = 9 CAR4 WT-treated mice). Two-tailed Mann–Whitney *U*-test was used for statistical analysis. **e**, Pro-B-DEVD cell tumors were incubated with 50 ng ml^−1^ IFN-γ and cell apoptosis was assessed by flow cytometry and expressed as a fold change relative to untreated cells. Each dot represents the mean of three technical replicates (from *n* = 2 independent experiments). **f**, Pro-B-cell tumors were incubated with IFN-γ (50 ng ml^−1^) or left untreated and subjected to live imaging. Images showing live tumor cells (magenta) and apoptotic tumor cells (blue). Scale bars, 20 μm. **g**, Images highlighting a pro-B-cell tumor undergoing apoptosis after 19 h of incubation with IFN-γ. Scale bar, 10 μm. Images are representative of *n* = 2 independent experiments. **h**, Tumor-bearing WT (left) and IFN-γ-R1^−/−^ (right) recipients were injected twice with 10 µg IFN-γ 24 h apart. Percentage of tumor cells (relative to untreated mice) in the bone marrow 2 d after the first IFN-γ injection. Data were compiled from *n* = 2 independent experiments (WT hosts, *n* = 7 untreated and *n* = 8 IFN-γ treated mice; IFN-γ-R1^−/−^ hosts, *n* = 7 untreated and *n* = 9 IFN-γ treated mice). Two-tailed Mann–Whitney *U*-tests were used for statistical analysis. **i**, Tumors were established in CD45.1^+^ mice by injection of a mixture of CD45.1^+^ IFN-γ-R1^+/+^ and CD45.2^+^ IFN-γ-R1^−/−^ pro-B cells. Six days later, mice were injected with CAR4 T cells. **j**, Tumor load (relative to untreated mice) was analyzed in the bone marrow (left), spleen (middle) and blood (right) 2–3 d after injection of CAR4 T cells. Data were compiled from *n* = 3 independent experiments. Each dot represents one mouse (*n* = 8 untreated and *n* = 11 CAR4 WT-treated mice). Two-tailed unpaired *t*-tests were used for statistical analysis. Data are expressed as mean ± s.e.m. ***P < 0.001; **P < 0.01; *P < 0.05; NS, not significant. Source data

As IFN-γ was essential for indirect killing events, we investigated the mechanism underlying tumor killing events observed at distance from CAR4 T cells. Regarding the long-range effects of IFN-γ, we envisioned at least two possibilities. First, we have previously shown that CAR4 T-cell-derived IFN-γ activates endogenous effectors such as T cells or NK cells by acting on host cells^15^ and these effectors could potentially contribute to the observed indirect killing events; however, indirect killing events were present at similar rate when CAR4 T cells were used to treat tumors in Prf1^−/−^ or WT recipients (Fig. 5a–c, Fig. 3c and Supplementary Video 5). This result suggests that perforin-dependent tumor killing by host cytotoxic effectors did not account for the indirect killing events. We also treated tumor-bearing IFN-γ-R1^−/−^ recipients with CAR4 T cells to test whether sensing of IFN-γ by host cells was important for antitumor activity in this model. CAR4 T cells effectively controlled tumor burden (Fig. 5d) suggesting that IFN-γ activity on host cells is dispensable for the regression of pro-B-cell tumors although a role at later time point to limit relapses cannot be excluded.

**Extended Data Fig. 6:**
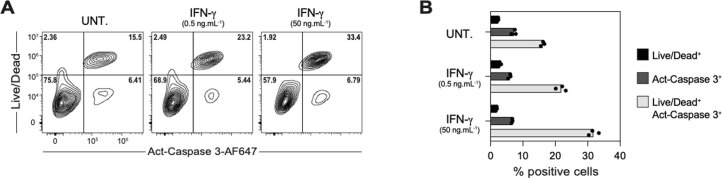
IFN-γ induces caspase 3 activation in pro-B cell tumors. (**a, b**) Pro-B cell tumors were incubated with IFN-γ (0.5 or 50 ng.mL^−1^) for 24 hours or left untreated. (**a**) Representative FACS plot and (**B**) bar graphs of cell death assessed using fixable viability dye (eFluor 780, eBioscience) and intracellular staining of activated caspase 3. Each dot represents one technical replicate (n = 1 experiment). Source data

**Extended Data Fig. 7:**
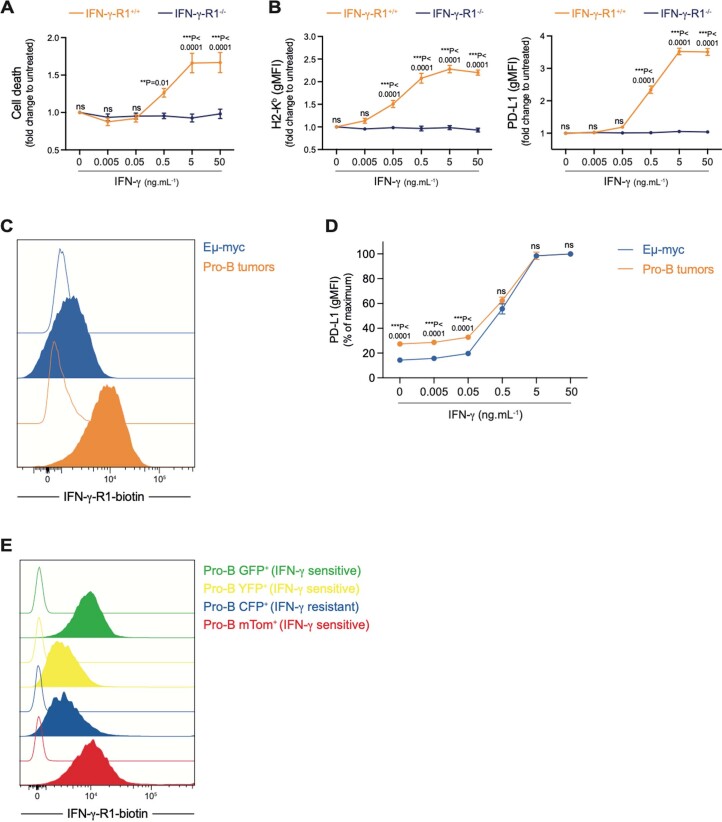
IFN-γ-R1 expression in the various B cell tumors. (**a–b**) Validation of IFN-γ-R1^−/−^ pro-B cell tumors. (**a**) Impact of IFN-γ-induced cell death on IFN-γ-R1^+/+^ and IFN-γ-R1^−/−^ pro-B cell tumors *in vitro*. Pro-B cell tumors were incubated with the indicated IFN-γ concentrations *in vitro* for 24 hours. Cell death was then assessed using fixable viability dye (Zombie NIR) and expressed as a fold change relative to untreated cells. (**b**) Pro-B cell tumors were incubated with the indicated IFN-γ concentrations *in vitro* for 24 hours. H2-K^b^ (left) and PD-L1 (right) surface expression was then analyzed by flow cytometry and expressed as a fold change relative to untreated cells. gMFI, geometric mean fluorescence intensity. (**a, b**) Data are pooled from n = 4 independent experiments. Two-way ANOVA and Sidak’s multiple comparisons were used for statistical analysis. (**c**) IFN-γ-R1 surface expression on Eµ-myc and pro-B cell tumors. Empty histograms represent the unstained control. (**d**) Eµ-myc and pro-B cell tumors respond to similar IFN-γ concentrations to upregulate PD-L1. Eµ-myc and pro-B cell tumors were incubated with the indicated IFN-γ concentrations *in vitro* for 24 hours. PD-L1 surface expression was then analyzed by flow cytometry. Data are pooled from n = 3 (Eµ-myc) and n = 5 (pro-B) independent experiments. gMFI, geometric mean fluorescence intensity. Two-way ANOVA and Sidak’s multiple comparisons were used for statistical analysis. (**e**) IFN-γ-R1 surface expression on GFP^+^, YFP^+^, CFP^+^ and mTom^+^ pro-B cell tumors. Note that the resistant CFP^+^ pro-B tumor cell line expressed similar levels of IFN-γ-R1 than the sensitive YFP^+^ pro-B tumor cell line. Empty histograms represent the unstained control. Data are expressed as mean ± SEM. ***P<0.001; **P<0.01; ns, not significant. Source data

A second possibility is that CAR4 T-cell-derived IFN-γ directly acts on tumor cells to mediate remote killing events. Consistent with this hypothesis, we found that pro-B-cell tumors underwent apoptosis in the presence of IFN-γ in vitro as measured by caspase 3 reporter and intracellular staining of activated caspase 3 (Fig. 5e–g and Extended Data Fig. 6). Moreover, direct administration of IFN-γ in tumor-bearing mice reduced tumor burden and this phenomenon was also observed in IFN-γ-R1^−/−^ recipients in which only tumor cells could sense IFN-γ (Fig. 5h). Finally, we specifically tested the role of IFN-γ sensing by tumor cells. To this end, we generated pro-B-cell tumors using IFN-γ-R1^−/−^ mice and confirmed their lack of response to IFN-γ exposure (Extended Data Fig. 7a,b). IFN-γ-R1^−/−^ tumors were mixed with fluorescent IFN-γ-R1 competent pro-B cells and co-injected in CD45.1^+^ congenic hosts (Fig. 5i and Extended Data Fig. 2b). CAR4 T cells displayed strong antitumor activity against IFN-γ-R1^+/+^ tumors but were ineffective against IFN-γ-R1^−/−^ tumors (Fig. 5j), indicating that IFN-γ sensing by tumor cells is essential for CAR4 T-cell activity. Altogether, these data strongly suggest that activity of CAR4 T-cell-derived IFN-γ on tumor cells is responsible for the observed indirect killing events.

**Extended Data Fig. 8:**
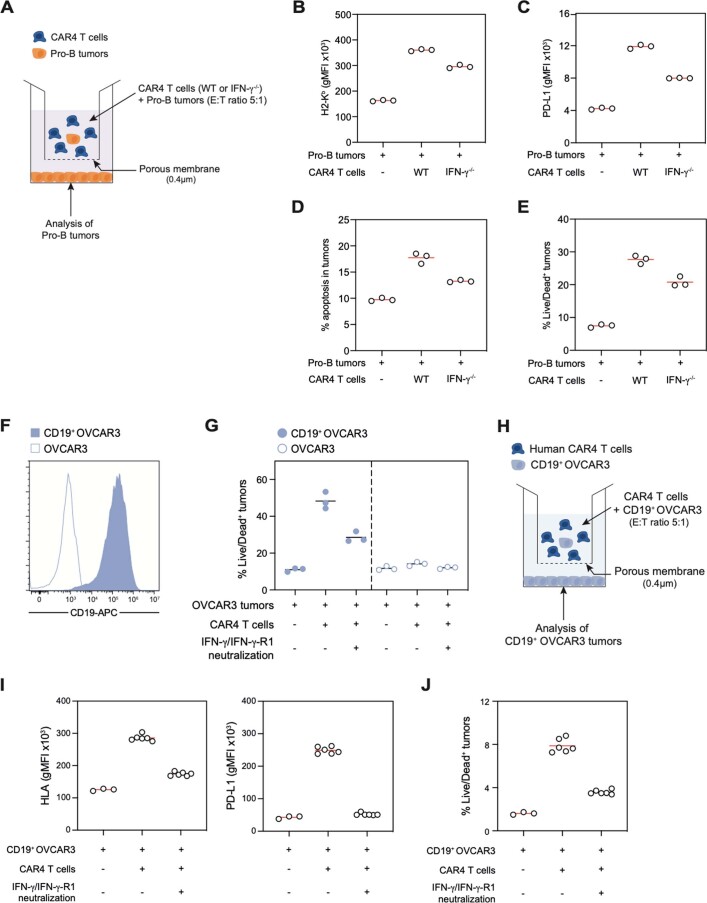
Mouse and human CAR4 T cells induce distant tumor killing in an IFN-γ-dependent manner. (**a**) Experimental setup. Pro-B tumors were co-cultured with WT or IFN-γ^−/−^ CAR4 T cells (at an effector-to-target (E:T) ratio 5:1) in the upper chamber of a transwell. Pro-B tumors (expressing the caspase 3 reporter) were added to the lower chamber. When indicated, IFN-γ was neutralized using a blocking antibody. 24 hours later, pro-B tumors located in the lower chamber were analyzed by flow cytometry. (**b, c**) Dot plots showing that CAR4 T cell-derived IFN-γ diffuses to induce the up-regulation of H2-K^b^ (**B**) and PD-L1 (**C**) levels on tumor cells. gMFI, geometric mean fluorescence intensity. (**d–e**) Dot plots showing that CAR4 T cell-derived IFN-γ induces distant tumor cell killing. (**d**) Tumor cell apoptosis was assessed using the FRET-based caspase 3 reporter. (**e**) Tumor cell death was assessed using fixable viability dye (Zombie NIR). (**b–e**) Each dot represents one technical replicate (n = 1 experiment). (**f**) Representative histogram of human CD19 expression by retrovirally transduced OVCAR3 tumors. (**g**) *In vitro* quantification of human CAR4 T cell cytotoxic activity. Antigen-negative (CD19^−^) OVCAR3 or antigen-positive (CD19^+^) OVCAR3 tumors were co-cultured with human anti-CD19 CAR4 T cells at 1:1 effector-to-target ratio or left untreated for 48 hours. When indicated, IFN-γ and IFN-γ-R1 were neutralized using blocking antibodies. Tumor cell death was assessed using fixable viability dye (Zombie NIR). Each dot represents one technical replicate (n = 1 experiment). (**h**) Experimental setup. CD19-expressing OVCAR3 cells were co-cultured with human CAR4 T cells (at an effector-to-target (E:T) ratio 5:1) in the upper chamber of a transwell. CD19-expressing OVCAR3 tumors were added to the lower chamber. When indicated, IFN-γ and IFN-γ-R1 were neutralized using blocking antibodies. 48 hours later, CD19-expressing OVCAR3 tumors located in the lower chamber were analyzed by flow cytometry. (**i**) Dot plots showing that CAR4 T cell-derived IFN-γ diffuses to induce the up-regulation of HLA molecules (left) and PD-L1 (right) levels on tumor cells. gMFI, geometric mean fluorescence intensity. (**j**) Dot plots showing that CAR4 T cell-derived IFN-γ induces distant tumor cell death as assessed using fixable viability dye (Zombie NIR). (**i, j**) Each dot represents one technical replicate. Similar results were obtained in n = 2 independent experiments. Source data

To confirm that CAR4 T cells could kill target cells at distance through IFN-γ production, we set up a Transwell assay in which CAR4 T cells and pro-B-cell tumors were placed in the upper chamber, while only tumor cells were present in the bottom chamber (Extended Data Fig. 8a). In this setup, we observed killing of the tumor cells in the bottom chamber, which was partly blocked when using IFN-γ^−/−^ CAR4 T cells (Extended Data Fig. 8b–e). To test whether long-range tumor killing can also be detected with human cells, we generated human anti-CD19 CAR4 T cells and transduced the human CD19 (hCD19) molecule in the human ovarian adenocarcinoma OVCAR3 tumor cell line (Extended Data Fig. 8f). As shown in Extended Data Fig. 8g, human CAR4 T cells effectively killed OVCAR3 tumors in an antigen-dependent manner, a process that was largely dependent on IFN-γ. Moreover, we confirmed using our Transwell assay that tumor killing by human CAR4 T cells can happen at distance in an IFN-γ-dependent manner (Extended Data Fig. 8h–j).

**Extended Data Fig. 9:**
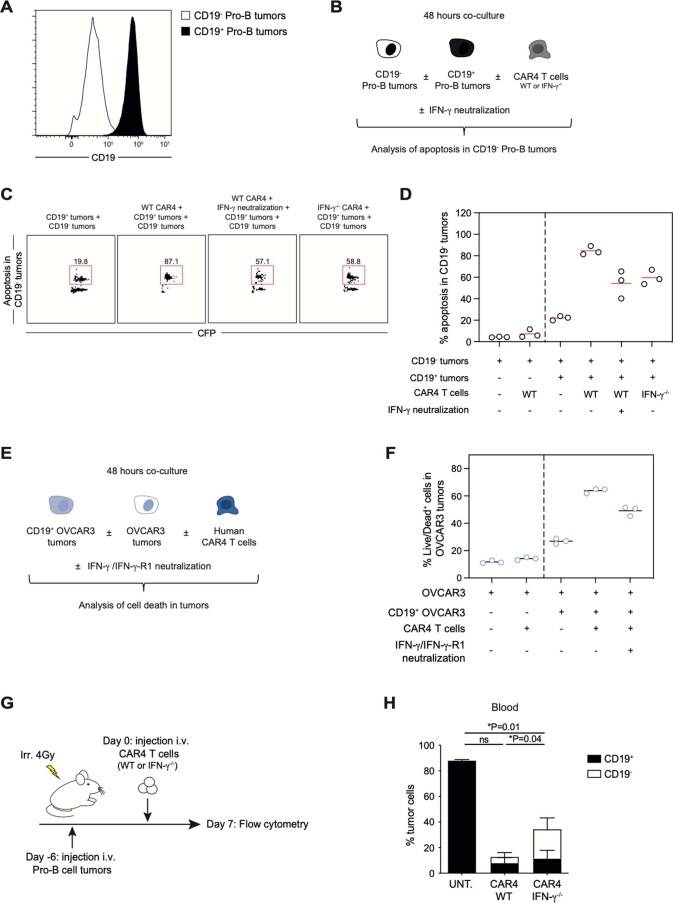
Murine and human CAR4 T cell-derived IFN-γ promotes the bystander killing of CD19^−^ target cells. (**a**) Representative histogram of CD19 expression by antigen-positive (black) and CRISPR-edited antigen-negative (white) pro-B cell tumors. (**b**) Experimental setup. Antigen-negative (CD19^−^) pro-B tumors were co-cultured with antigen-positive (CD19^+^) pro-B tumors and WT or IFN-γ^−/−^ CAR4 T cells. When indicated, IFN-γ was neutralized using a blocking antibody. 48 hours later, apoptosis in CD19^−^ pro-B tumors (expressing the caspase 3 reporter) was analyzed by flow cytometry. (**c, d**) Representative FACS plots (**C**) and dot plots (**D**) showing that CAR4 T cell-derived IFN-γ produced upon recognition of CD19^+^ pro-B tumors contributes to the apoptosis of CD19^−^ pro-B tumors. Each dot represents one technical replicate (n = 1 experiment). CFP, Cyan Fluorescent Protein. (**e**) Experimental setup. Antigen-negative (CD19^−^) OVCAR3 tumors were co-cultured with antigen-positive (CD19^+^) OVCAR3 tumors and human anti-CD19 CAR4 T cells. When indicated, IFN-γ and IFN-γ-R1 were neutralized using blocking antibodies. 48 hours later, cell death in OVCAR3 tumors was analyzed by flow cytometry using fixable viability dye (Zombie NIR). (**f**) Dot plots showing that human CAR4 T cell-derived IFN-γ produced upon recognition of CD19^+^ OVCAR3 tumors contributes to the killing of antigen-negative OVCAR3 tumors. Each dot represents one technical replicate (n = 1 experiment). (**g**) *In vivo* experimental set-up. Pro-B cell tumors were established by intravenous injection of 0.5 × 10^6^ of pro-B cells expressing the FRET-based caspase 3 reporter in C57BL/6 mice after sublethal irradiation. Six days later, mice were injected intravenously with WT or IFN-γ^−/−^ CAR4 T cells or left untreated. Seven days after CAR T cell transfer, blood cells were processed using flow cytometry. (**h**) Percentage of antigen-positive (CD19^+^) and emerging antigen-negative (CD19^−^) pro-B tumors tumor cells recovered from the blood (n = 7 untreated, n = 8 WT CAR4 T cell-treated and n = 8 IFN-γ^−/−^ CAR4 T cell-treated mice from 1 experiment). Statistics for antigen-negative (CD19^−^) pro-B tumors are shown. Two-way ANOVA and Tukey’s multiple comparisons were used for statistical analysis. Data are expressed as mean ± SEM. *P<0.05; ns, not significant. Source data

Our observations raised the possibility that CAR4 T-cell-derived IFN-γ may also act on CD19^−^ tumors, possibly limiting antigen-escape variants. We first tested this idea in vitro by mixing CD19^+^ pro-B cells with CD19^−^ pro-B cells (generated by CRISPR/Cas9) (Extended Data Fig. 9a,b). We observed bystander killing of CD19^−^ targets by CAR4 T cells that was reduced upon neutralization of IFN-γ or when using IFN-γ^−/−^ CAR4 T cells (Extended Data Fig. 9c,d). Indirect killing required the presence of CD19^+^ targets in the culture, most likely to initiate IFN-γ production. Similar observations were made using human CAR4 T cells and a mixture of CD19^−^ and CD19^+^ OVCAR3 tumors (Extended Data Fig. 9e,f). Second, we tested the emergence of CD19^−^ pro-B-cell tumors in vivo after treatment with WT or IFN-γ^−/−^ CAR4 T cells (Extended Data Fig. 9g). As shown in Extended Data Fig. 9h, we observed a reduced frequency of CD19^−^ pro-B-cell tumors after treatment with WT as compared to IFN-γ^−/−^ CAR4 T cells. These results revealed a potential role for CAR4 T-cell-derived IFN-γ in limiting the emergence of antigen-negative tumors.

### CAR4 T cells selectively eliminate IFN-γ-sensitive tumors

**Extended Data Fig. 10:**
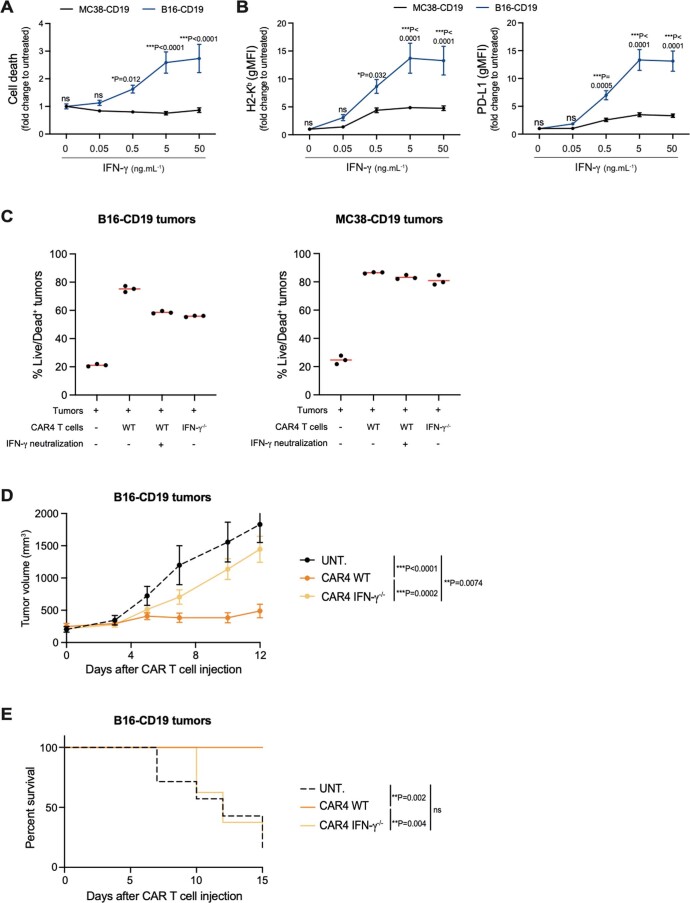
IFN-γ contributes to CAR4 T cell-mediated killing of some but not all solid tumors. (**a, b**) Impact of IFN-γ on two distinct solid tumors, colon adenocarcinoma MC38 cells and melanoma B16.F10 cells. MC38 and B16.F10 tumors were incubated with the indicated IFN-γ concentrations *in vitro* for 24 hours. (**a**) Cell death was assessed using fixable viability dye (Zombie NIR) and expressed as a fold change relative to untreated cells. (**b**) IFN-γ induces phenotypic changes in both MC38 and B16.F10 tumors *in vitro*. H2-K^b^ (left) and PD-L1 (right) surface expression was then analyzed by flow cytometry. gMFI, geometric mean fluorescence intensity. (**a, b**) Each dot represents the mean of 3 technical replicates from n = 2 independent experiments. Two-way ANOVA and Sidak’s multiple comparisons were used for statistical analysis. (**c**) CD19-expressing MC38 and CD19-expressing B16.F10 tumors were co-cultured with WT or IFN-γ^−/−^ CAR4 T cells at 1:1 effector-to-target ratio or left untreated for 24 hours. When indicated, IFN-γ was neutralized using a blocking antibody. Tumor cell death was assessed using fixable viability dye (Zombie NIR). Each dot represents one technical replicate (n = 1 experiment). (**d-e**) B16-CD19 tumors were established by subcutaneous injection of 0.5 × 10^6^ B16-CD19 cells in the flank of C57BL/6 mice after sublethal irradiation. Ten days later, mice were injected intravenously with WT or IFN-γ^−/−^ CAR4 T cells (10 × 10^6^) or left untreated (n = 7 untreated mice, n = 7 WT CAR4 T cell-treated mice and n = 8 IFN-γ^−/−^ CAR4 T cell-treated mice from 1 experiment). (**d**) Tumor growth was monitored every 2–3 days. Two-way ANOVA and Tukey’s multiple comparisons were used for statistical analysis. (**e**) WT CAR4 but not IFN-γ^−/−^ CAR4 T cell therapy prolonged mouse survival. Log-rank test was used for statistical analysis. Data are expressed as mean ± SEM. ***P<0.001; **P<0.01; *P<0.05; ns, not significant. Source data

Distinct tumors exhibit variable sensitivity to IFN-γ-induced cell death^44,46–48^. We therefore asked whether the sensitivity to IFN-γ-induced cell death dictate the antitumor potential of CAR4 T cells. In line with this idea, we found that Eµ-myc B-cell tumors that are insensitive to CAR4 T-cell therapy did not undergo cell death in the presence of IFN-γ^15,44^. Differences in IFN-γ sensitivity were also observed in solid tumors (Extended Data Fig. 10a,b). We found that IFN-γ triggers the cell death of B16 melanoma but not MC38 colon carcinoma cells. Consistently, we found a contribution of IFN-γ in CAR4 T-cell-mediated killing in vitro only for B16-CD19 tumors but not for MC38-CD19 tumors (Extended Data Fig. 10c). In vivo, we observed that WT but not IFN-γ^−/−^ CAR4 T cells controlled the growth of B16-CD19 tumors and increased survival, extending the importance of CAR4 T-cell-derived IFN-γ to a solid tumor model (Extended Data Fig. 10d,e).

**Fig. 6:**
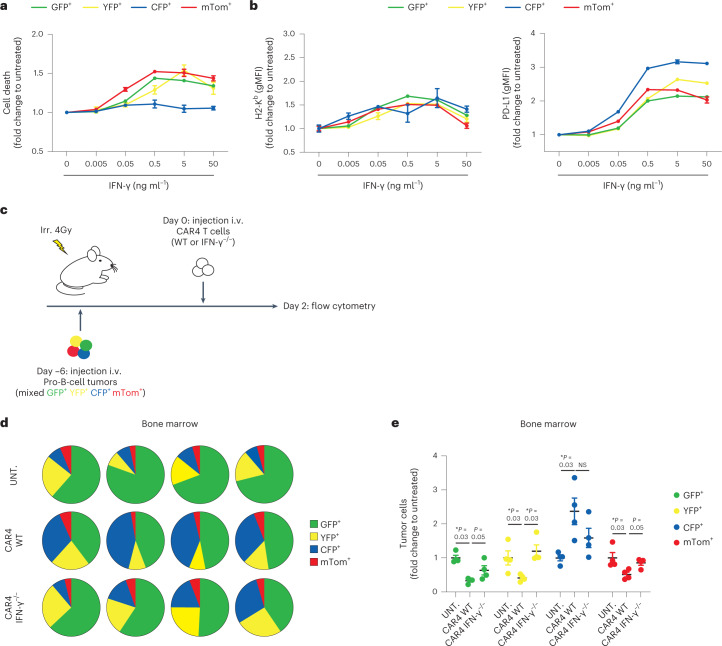
CAR4 T cells selectively eliminate IFN-γ-sensitive tumor cells. **a**,**b**, Impact of IFN-γ on different pro-B-cell tumors in vitro. Distinct, independently generated, pro-B-cell tumors were incubated with the indicated IFN-γ concentrations in vitro for 24 h. Cell death was assessed using Fixable Viability Dye (Zombie NIR) and expressed as a fold change relative to untreated cells (**a**). Each dot represents the mean of three technical replicates. IFN-γ induces phenotypic changes in all pro-B-cell tumors in vitro (**b**). Pro-B-cell tumors were incubated with the indicated IFN-γ concentrations in vitro for 24 h. H2-K^b^ (left) and PD-L1 (right) surface expression was then analyzed by flow cytometry. Each dot represents the mean of three technical replicates. gMFI, geometric mean fluorescence intensity. **c**, In vivo experimental setup. Pro-B-cell tumors were established by intravenous injection of 0.8 × 10^6^ of color-coded pro-B cells (1:1:1:1 ratio of a mix of CFP^+^GFP^+^YFP^+^mTom^+^ pro-B cells) in C57BL/6 mice after sublethal irradiation. Six days later, mice were injected intravenously with WT or IFN-γ^−/−^ CAR4 T cells. Two days after CAR T-cell transfer, bone marrow cells were processed using flow cytometry. **d**, Pie charts showing the distribution of individual colored pro-B tumors in the bone marrow two days after injection of CAR4 T cells. Each pie chart represents one mouse. **e**, Tumor cellular composition (fold change relative to untreated mice) was analyzed in the bone marrow 2 d after injection of CAR4 T cells. Each dot represents one mouse. Two-tailed Mann–Whitney *U*-tests were used for statistical analysis. Data shown in **a**–**e** are representative of *n* = 2 independent experiments (*n* = 4 mice per group). Data are expressed as mean ± s.e.m. *P < 0.05; NS, not significant. Source data

As Myc-driven and Abl-driven B-cell tumors likely differ by other factors, we also generated multiple fluorescent pro-B-cell tumors by viral Abl overexpression. Individually generated tumor cell lines are expected to harbor different genomic alterations and mutations^49^. Investigating seven different pro-B-cell lines, we found that all responded to IFN-γ exposure by upregulating MHC class I molecules and PD-L1 but only five showed substantial cell death (referred to as sensitive cell lines). We selected three sensitive (expressing green fluorescent protein (GFP), yellow fluorescent protein (YFP) or mTom) and one resistant (expressing cyan fluorescent protein (CFP)) cell lines (Fig. 6a,b) that were differently labeled with fluorescent proteins and mixed at equivalent proportion (Fig. 6c). Mice receiving this mixture were treated with WT or IFN-γ^−/−^ CAR4 T cells or left untreated. All four cell lines were detected in untreated mice although at different frequencies (Fig. 6d and Extended Data Fig. 2c), possibly due to small differences in growth rates. As shown in Fig. 6e, the contribution of each color-coded cell line (normalized to the percentage seen in untreated recipients) was modified by CAR4 T-cell treatment. All three sensitive tumor cell lines were efficiently controlled by CAR4 T cells in a manner that was largely dependent on CAR4 T-cell-derived IFN-γ. Notably, IFN-γ-resistant B-cell tumors were over-represented after CAR4 T-cell therapy (Fig. 6e). We tested whether IFN-γ-R levels may influence tumor sensitivity to IFN-γ-induced cell death. While Eμ-myc cells expressed lower IFN-γ-R levels than pro-B-cell tumors (Extended Data Fig. 7c), both responded to similar concentration of IFN-γ for PD-L1 upregulation (Extended Data Fig. 7d). In addition, there was no obvious correlation between IFN-γ-R levels and sensitivity to IFN-γ-induced cell death in the various Abl-driven pro-B-cell lines generated (Extended Data Fig. 7e and Fig. 6a), suggesting the contribution of other factors to the tumor responses to IFN-γ. In sum, these experiments therefore suggest that CAR4 T cells selectively eliminate tumor cells sensitive to IFN-γ-induced cell death but spare IFN-γ-resistant tumors.

### IFN-γ predicts survival in patients with high CAR4 T cells

**Fig. 7:**
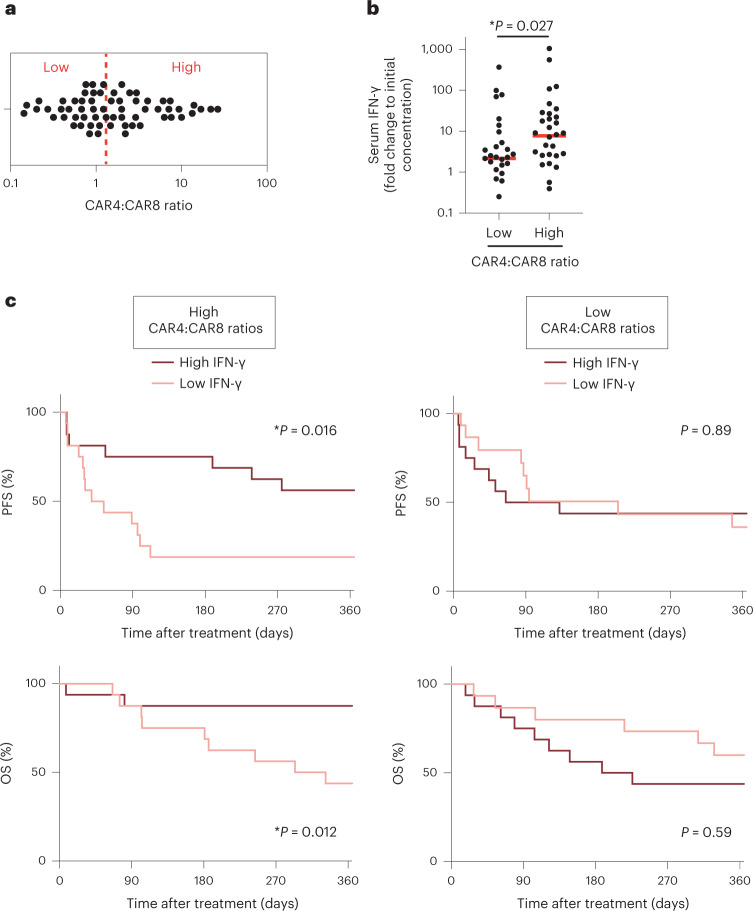
High IFN-γ induction is associated with improved survival in patients with DLBCL exhibiting high CAR4:CAR8 T-cell ratios. A cohort of patients with DLBCL (*n* = 63) treated with anti-CD19 CAR T cells at Saint-Louis Hospital (Paris) was analyzed for CAR4:CAR8 T-cell ratio in the blood (day 7 after CAR T-cell transfer) and for IFN-γ concentration in the serum (day 0 and day 7 after CAR T-cell transfer). **a**, Distribution of the CAR4:CAR8 T-cell ratios in the blood of treated patients. Each dot represents one patient (*n* = 63). Red dashed bar represents the median (1.31). **b**, Stronger IFN-γ induction in patients with high CAR4:CAR8 ratios. Scatter-plot showing the serum concentration of IFN-γ (expressed as a fold change relative to the baseline concentration measured at day 0) as a function of the ratio of CD4^+^ and CD8^+^ CAR T cells. Each dot represents one patient (*n* = 63). Red bars represent the median. Two-tailed Mann–Whitney *U*-test was used for statistical analysis and associated *P* value is shown. **c**, Induction of IFN-γ correlates with improved clinical outcome in patients with high CAR4:CAR8 ratios. Kaplan–Meier curves showing 1-year estimates of PFS (top) and OS (bottom) for patients displaying high (above the median) CAR4:CAR8 ratios (left) or low (below the median) CAR4:CAR8 ratios (right) as a function of IFN-γ induction. The median of IFN-γ induction (7.72 for patients with high CAR4:CAR8 ratios and 2.16 for patients with low CAR4:CAR8 ratios) was used to discriminate patients with low IFN-γ or high IFN-γ. Log-rank tests were used for statistical analysis and *P* values are shown. Source data

Our mouse data suggested that IFN-γ production is particularly important for optimal CAR4 T-cell antitumor activity. To test the relevance of these findings in humans, we analyzed a cohort of 63 anti-CD19 CAR T-cell-treated patients with diffuse large B-cell lymphoma (DLBCL), monitoring both CAR4:CAR8 T-cell ratio in the blood and serum concentration of IFN-γ 1 week post-transfer (around the peak of CAR T-cell expansion). As previously reported, we noted extensive differences in CAR4:CAR8 T-cell ratios among patients (Fig. 7a). Notably, we observed that patients with high (above the median) CAR4:CAR8 T-cell ratios exhibited stronger induction of IFN-γ (Fig. 7b). This observation is reminiscent of the higher levels of circulating IFN-γ seen in our mouse model treated with CAR4 T cells compared to CAR8 T cells (Fig. 4a). Most notably, we analyzed the progression-free survival (PFS) and overall survival (OS) of CAR T-cell-treated patients depending on their CAR4:CAR8 ratio and IFN-γ induction after CAR T-cell transfer. In patients with high CAR4:CAR8 ratios, high IFN-γ induction (above the median) was associated with significantly improved PFS and OS (Fig. 7c). By contrast, IFN-γ induction was not predictive of treatment outcome in patients with low CAR4:CAR8 ratios (Fig. 7c). These clinical data support the idea that IFN-γ is a hallmark of potent antitumor activity mediated by the CD4^+^ subset of CAR T cells.

## Discussion

CD4^+^ T cells have been shown to exert potent antitumor activity in preclinical and clinical settings^1–9^ but we lack direct evidence of how this activity is achieved. Here, we addressed the mechanisms underlying the antitumor activities of CAR4 T cells. We found that when used alone CAR4 T cells can be highly effective at mediating tumor regression in some models but not others. Mechanistically, CAR4 T cells eliminated sensitive tumor cells at distance by forming large IFN-γ fields in the tumor microenvironment. Our results support the idea that the intrinsic sensitivity of tumor cells to the pro-apoptotic effects of IFN-γ is a major determinant of CAR4 T-cell therapy.

CAR4 T cells can exert antitumor activity through multiple mechanisms but the exact contribution of these mechanisms to tumor cell elimination in vivo has remained technically challenging to quantify. The ability to probe tumor apoptosis in real-time using intravital imaging provided us with a unique means to quantify killing events and their dependence on cell contact with CAR4 T cells. Notably, CAR4 T-cell killing through cell–cell interaction accounted for less than a third of total tumor apoptotic events and this killing mode was largely dependent on perforin. Most indirect killing events were independent on perforin but dependent on the capacity of CAR4 T cells to produce IFN-γ and trigger tumor apoptosis at distance. Consistently, this killing mode was essential for the therapeutic benefit of CAR4 T cells in this model.

Recent studies that used a mixture of CAR4 and CAR8 T cells have observed that IFN-γ is dispensable for killing of hematologic tumors^50,51^. This finding is not incompatible with our observation as the requirement for IFN-γ described in our study pertains specifically to CAR4 T cells (but not CAR8 T cells). It has been recently shown that IFN-γ can increase contact-dependent killing of CAR T cells by upregulating adhesion molecules on tumor cells such as ICAM-1 (refs. ^51,52^) or by sustaining CAR T-cell cytotoxic functions^15^. We described here a distinct mechanism whereby IFN-γ produced by CAR4 T cells acts remotely to induce apoptosis, independently of cell contact and of the perforin pathway.

We and others have observed markedly different outcomes when distinct B-cell tumors were treated with CAR4 T cells^15–17,19,21,23^. We provide evidence that the pro-apoptotic effects of IFN-γ were essential for robust CAR4 T-cell activity. Given that mouse or human tumors display highly variable sensitivities to IFN-γ-mediated cell death^44,46–48^, our results likely provide an explanation for these discrepancies. In fact, by artificially mixing IFN-γ-sensitive and IFN-γ-resistant tumors, we observed outgrowth of resistant cells upon CAR4 T-cell therapy, possibly reflecting the emergence of tumor cells with mutations in the IFN-γ pathway observed in preclinical models^53,54^ or in patients treated by immunotherapy^55^. This is not to mean that CAR4 T cells are useless for tumors insensitive to IFN-γ-induced cell death. In fact, when used in combination with CAR8 T cells, CAR4 T cells could also sustain CAR8 T-cell cytotoxicity through IFN-γ production and crosstalk with the tumor microenvironment as we previously reported^15^.

Several factors have been shown to contribute to tumor cell death in response to IFN-γ, including the expression levels of IRF1 (refs. ^46,56^), IFN-γ-R^57,58^, SLFN11 (ref. ^47^), SOCS1 (ref. ^59^), Fas/Fas ligand^60^, TRAIL^61^, iNOS^62^ and the production of reactive oxygen species and reactive nitrogen intermediates^63^. These factors together with the functional response of tumor cells to IFN-γ may represent interesting biomarkers to identify patients who would most benefit from a CAR4 T-cell-enriched infusion product. Indeed, while most patients treated by anti-CD19 CAR T cells are infused with a mixture of CAR4 and CAR8 T cells at very variable ratios^11,12,14^, each subset seems endowed with some unique functional capabilities. We have previously observed that CAR4 T cells are generally more effective than CAR8 T cells at activating host effector cells such as CD8^+^ T cells and NK cells^15^ and boosting the host immune response may help counteract the emergence of antigen-negative escape variants. In addition, it has been proposed that CAR4 T cells may be less prone to exhaustion as compared to CAR8 T cells^19^. Finally, while both CAR4 and CAR8 T cells can produce IFN-γ, we found the production by CAR4 T cells to be >30 fold higher than that mediated by CAR8 T cells in our model. Notably, we observed that CAR T-cell-treated patients with DLBCL with high CAR4:CAR8 ratios were also more prone to display high levels of IFN-γ after CAR T-cell transfer. Induction of IFN-γ upon CAR T-cell transfer was associated with a better clinical outcome, but selectively for patients displaying high CAR4:CAR8 ratios. These data may suggest that IFN-γ is critical for CAR4 T-cell antitumor activity in humans. Given the importance of IFN-γ diffusion in the tumor microenvironment^44,45^, CAR4 T cells should largely contribute to the generation of large cytokine fields in the tumor microenvironment that can act at long-distance to eliminate IFN-γ-sensitive tumor cells, possibly including antigen-loss variants as demonstrated herein.

As exemplified here, understanding how tumor-intrinsic factors influence mechanisms of CD4^+^ T-cell activity represents an important step for leveraging the unique contributions of CD4^+^ and CD8^+^ CAR T cells for personalized medicine.

## Methods

### Ethics statement

Animal studies were approved by the Safety Committee of Institut Pasteur and in accordance with European and French guidelines (CETEA 2017-0038). The maximal tumor size for animal experiments based on solid tumors was 2,000 mm^3^ and 500 mm^3^ for experiments based on liquid tumors. Maximal tumor size was not exceeded. The institutional review-board of Saint-Louis Hospital (BIOCART-CPP 2019-77) approved this study and all patients signed informed consent.

### Mice and cell lines

C57BL/6J mice were obtained from Envigo. *Ifng*^−/−^, *IfngR1*^−/−^, *Prf1*^−/−^, *ROSA*^mT/mG^, *Ubi-GFP*, *CFP* and *CD45.1* mice were bred and crossed in our animal facility under specific-pathogen-free conditions, air renewal (20 times per hour), constant ambient temperature of 22 ± 2 °C and 14:10 h light–dark cycle. All mice used were aged 6–8 weeks old. Male mice were used when using male tumor cell lines. Female mice were used when using female tumor cell lines. The lymphoma B-cell tumor was isolated from a male Eμ-myc mouse, a transgenic mouse line developing spontaneous Burkitt-like lymphomas^64^. Immortalized pro-B cells were obtained by transducing bone marrow cells with a retrovirus encoding viral-Abelson kinase (v-abl)^65^. These cell lines were retrovirally transduced to express a reporter for caspase 3 activity (named Eµ-myc-DEVD or Pro-B-DEVD)^66^, YFP or STAT1–GFP fusion protein. Immortalized pro-B cells expressing tdTomato, CFP or GFP were similarly generated by infecting bone marrow cells of ROSA^mT/mG^, CFP and Ubi-GFP mice, respectively. Immortalized pro-B cells deficient for IFN-γ sensing were generated by infecting bone marrow cells of IFN-γ-R1^−/−^ mice. B16.F10 melanoma (ATCC) and MC38 colon adenocarcinoma (Kerafast) cells were retrovirally transduced to express the murine CD19 protein. OVCAR3 ovarian adenocarcinoma cells (ATCC) were retrovirally transduced to express the human CD19 protein. HEK cells (ATCC) were purchased at ATCC. Mice were examined every day and killed in case of prostration, weakness, tousled hair or nodal tumor mass >1 cm. Cells were cultured in RPMI medium 1640-GlutaMAX or DMEM high glucose GlutaMAX supplemented with 10% heat-inactivated fetal bovine serum, 50 U ml^−1^ penicillin, 50 μg ml^−1^ streptomycin, 1 mM sodium pyruvate, 10 mM HEPES and 50 μM 2-mercaptoethanol, and maintained at 37 °C and 5% CO_2_. All cell lines were regularly tested for the lack of *Mycoplasma* contamination using the Venor-GeM Advance *Mycoplasma* detection kit (Minerva Biolabs).

### CAR T-cell generation and adoptive transfer

The retroviral vector encoding anti-CD19 CAR (tCD34.2A.amCD19.CD28IEVζ) has been previously described^67^. In brief, the CAR construct comprises the anti-mouse CD19 single-chain fragment variable domain from the 1D3 rat hybridoma, the transmembrane and intracellular domains of CD28 and the CD3ζ intracellular domain. The vector also encodes a truncated form of the human CD34 molecule used for the identification and isolation of CAR-expressing T cells. CD4^+^ and CD8^+^ T cells were purified from spleens and lymph nodes of WT, *Ifng*^−/−^ and *Prf1*^−/−^ male mice using either CD4-negative or CD8-negative selection kits (Miltenyi Biotec). T cells were stimulated using plates coated with anti-CD3 monoclonal antibodies in the presence of soluble anti-CD28 monoclonal antibody and 10 ng ml^−1^ murine IL-12 (I8523; Sigma-Aldrich)^68^. T cells were then subjected to two rounds of spin-infections (performed at 24 and 48 h) using retroviral supernatant and 8 μg ml^−1^ polybrene (Merck). T cells were cultured for 4 d in the presence of 10 ng ml^−1^ hIL-2 (202-IL; R&D Systems). For in vivo imaging experiments, CAR T cells were retrovirally transduced to express GFP. For comparable in vitro activation, IFN-γ-deficient T cells and their WT counterparts were supplemented with 100 ng ml^−1^ of IFN-γ (Peprotech). CAR transduction efficacy was routinely >80%; in cases of lower efficacy, transduced cells were isolated using the hCD34 positive selection kit (Miltenyi Biotec). Tumors were established by injecting 0.5 × 10^6^ Eμ-myc cells or 0.5–0.8 × 10^6^ transformed pro-B cells in mice following a conditioning regimen (4 Gy sublethal irradiation) to promote CAR T-cell engraftment^69^. B-cell tumors developed initially in the bone marrow and became detectable in the blood by day 6–7. At this time, CAR T cells (5–20 × 10^6^ cells) were injected i.v. For the solid tumor model, tumors were established by injecting 0.5 × 10^6^ B16-CD19 cells subcutaneously in the flank of sublethally irradiated (4 Gy) mice. Tumors were allowed to grow for 10 d at which time CAR T cells (10 × 10^6^ cells) were injected intravenously. Starting tumor volumes were normalized between treatment groups. Tumor growth was measured every 2–3 d using a caliper.

Human CD4^+^ CAR T cells were generated from peripheral blood mononuclear cells from healthy donors obtained from Etablissement Français du Sang. All donors gave informed consent. CD4^+^ T cells were isolated using the CD4^+^ T-cell isolation kit (Miltenyi Biotec) and stimulated with Dynabeads Human T-Activator CD3/CD28 (Thermo Fisher Scientific) in X-VIVO 15 medium (Lonza) supplemented with 5% human serum (Sigma-Aldrich) and 50 μM 2-mercaptoethanol. Twenty-four hours after activation, CD4^+^ T cells were transduced with lentiviral supernatants corresponding to an anti-CD19(FMC63)-CD8tm-CD28-CD3ζ CAR (rLV.EF1.19(FMC63)-28z, Flash Therapeutics) at a multiplicity of infection of 20. After two additional days, CD3/CD28 beads were magnetically removed and CD4^+^ CAR T cells were used for in vitro assays.

### Generation of IFN-γ-R1^−/−^ pro-B cells

A Pro-B-DEVD cell line lacking IFN-γ-R1 was generated by genome editing of Pro-B-DEVD cell tumors with CRISPR/Cas9 using the Alt-R CRISPR-Cas9 System from Integrated DNA Technologies (IDT) and following manufacturer’s recommendations. The sequence used to target IFN-γ-R1 was ATGTGGAGCATAACCGGAGT. Briefly, CRISPR-Cas9 crRNA and CRISPR-Cas9 tracrRNA were purchased from IDT. To form active guide RNA complexes, an equimolar concentration of crRNA and tracrRNA (1 µM each) were hybridized by heating up to 95 °C for 5 min and allowed to cool slowly to 23 °C in a PCR thermocycler. CRISPR–ribonucleoprotein (RNP) complexes were formed by mixing 1 µg S.p. HiFi Cas9 Nuclease V3 (IDT) to gRNA for 10 min at room temperature. One million pro-B-cell tumors were resuspended in 20 µl of nucleofection solution with 3 µl RNP and transferred to nucleocuvette strips (SF Cell Line 4D-Nucleofector X Kit S, Lonza) for electroporation using the DN-110 program of 4D-Nucleofector X Unit. Electroporated cells were cultured for 3 d at 32 °C in 5% CO_2_ to allow genome editing. Effective IFN-γ-R1 ablation was confirmed by flow cytometry.

### IFN-γ treatments

For in vitro IFN-γ exposure on tumor cells, different IFN-γ (recombinant murine IFN-γ; Peprotech or recombinant human IFN-γ; Peprotech) concentrations were used ranging from 0.005–1,000 ng ml^−1^ for the indicated time. For in vivo cytokine supplementation studies, murine IFN-γ (10 μg in 100 μl sterile PBS) was delivered twice i.v. for 2 d starting 7–8 d after tumor inoculation.

### Transwell assay

To assess the ability of IFN-γ to diffuse and induce tumor killing at distance, a Transwell assay was set up. Tumor cells and CAR T cells were co-cultured in the upper chamber of a 24-well Transwell plate (0.4-µm pore polycarbonate membrane insert; Corning). Tumor cells were added to the lower chamber. At 24–48 h later, tumor cells from the lower chamber were collected and analyzed using flow cytometry. When indicated, IFN-γ and IFN-γ-R1 were neutralized by adding blocking antibodies in the top and bottom chambers.

### Multiplex assay for cytokine quantification

Sera were isolated from blood obtained by cardiac puncture 3 d after CAR T-cell transfer and snap frozen before storage at −20 °C until analysis. Multiplex cytokine assay was performed using 26-Plex ProcartaPlex Panel (Invitrogen). Plates were incubated overnight at 4 °C under agitation and analyses were performed using a Bio-Plex 200 system and the Bio-Plex Manager software (Bio-Rad).

### Flow cytometry and antibodies

Bone marrow cells were obtained from tibias and femurs and filtered using 70-µm cell strainers. Spleen cells were filtered through 70-µm cell strainers. After killing the mice, blood was collected by cardiac puncture and treated with a red blood cell lysis buffer (eBiosciences). When indicated, cells were stained with live/dead (Fixable Viability Dye eFluor 780 (eBioscience) or Zombie NIR Fixable Viability kit (BioLegend)) staining during 20 min at room temperature. Then single-cell suspensions were Fc-blocked using anti-CD16/32 monoclonal antibodies and normal murine serum 1%. Staining was performed with the following monoclonal antibodies: hCD34-PE or Alexa Fluor 647, hCD19-APC, hHLA-A,B,C-BV605, hPD-L1-PE, CD4-BUV395 or BV786, CD8a-BUV395, CD11b-BUV395, CD19-PE/Cy7 or APC-fire750, CD44-Alexa488, CD45.1-PE, CD45.2-BUV737, CD62L-BV421, H-2Db-APC, H-2Kb-PE/Cy7, LAG-3-APC-fire750, PD-1-PE/Cy7, PD-L1-APC or BV785, TIGIT-PE and Tim-3-BV785. For surface ICAM-1 and IFN-γ-R1 staining, cells were stained with biotin-conjugated anti-ICAM-1 monoclonal antibody or biotin-conjugated anti-CD119 (IFN-γ-R1) monoclonal antibody, respectively and then PE-conjugated streptavidin or APC-fire750-conjugated streptavidin. Intracellular staining was performed using the Cytofix/Cytoperm kit (BD Biosciences) according to the manufacturer’s guidelines and Alexa647-conjugated anti-active caspase 3 monoclonal antibody. Analyses were performed using a Cytoflex LX (Beckγman Coulter) flow cytometer, CytExpert 2.3 software (Beckman Coulter) and analyzed with FlowJo v.10.8.1 (BD). Tumor cell apoptosis was quantified using a FRET-based reporter for caspase 3 activity. FRET loss was defined as new parameter using the ratio of CFP to FRET fluorescence. FRET loss was quantified after fixing cells with 2% paraformaldehyde solution (Sigma) directly after ex vivo isolation. Murine IFN-γ was blocked using 50 µg ml^−1^ of purified anti-mouse IFN-γ antibody. Human IFN-γ and IFN-γ-R1 were blocked using 50 µg ml^−1^ of purified mouse anti-human IFN-γ and 25 µg ml^−1^ of anti-human IFN-γ-R1 antibody, respectively. For TRAIL and FasL neutralization, 2 µg ml^−1^ of anti-mouse CD253 (TRAIL) antibody and 10 µg ml^−1^ of purified anti-mouse/rat CD178 (FasL) antibody were used, respectively. Further information on antibody clones, dilutions and providers is available in the Nature Research Reporting Summary linked to this article.

### In vitro imaging

Plastic dishes were coated with poly-d-lysine (Sigma, 0.01% dilution in PBS) for 30 min at 37 °C. Cells were incubated in the culture dishes in complete RPMI without phenol red containing or not IFN-γ (50 ng ml^−1^) for the indicated time before being subjected to imaging. In vitro two-photon imaging was performed using an upright microscope (FVMPE-RS, Olympus), a ×25/1.05 numerical aperture, water-dipping objective combined with an objective heater and using FV31S-SW software (Olympus). An Insight DeepSee dual laser (Spectra-Physics) tuned at 880 nm was used for excitation. The following filters were used: CFP (483/32) and YFP (542/27). Timelapse sequences were typically created by scanning a 25-μm-thick tissue volume using 5-μm z-steps and 30-s intervals.

### Intravital two-photon imaging of the bone marrow

Bone marrow in vivo imaging was performed 2–3 d after CAR T-cell treatment^42^. During imaging experiments, mice were supplied with oxygen and temperature was kept at 37 °C using a heated pad. Two-photon imaging was performed with an upright microscope (FVMPE-RS, Olympus), a ×25/1.05 NA water-dipping objective equipped with an objective heater and using FV31S-SW software (Olympus). An Insight DeepSee dual laser (Spectra-Physics) tuned at 860 or 880 nm was used for excitation. The following filters were used: CFP (483/32), GFP (520/35 or 512/25), YFP (542/27) and background (593/40). Timelapse sequences were created by scanning a 30–40-μm-thick tissue volume using 5-μm z-steps and 60-s intervals.

### Image analysis

Videos were processed and analyzed using Fiji software (ImageJ v.2.3.0). Figures and videos based on two-photon microscopy are two-dimensional maximum intensity projections of three-dimensional data. Tumor apoptotic events were quantified manually and scored as direct killing when a CAR T cell interacted with the tumor cell before the detection of FRET loss or as indirect killing when tumor cells underwent FRET loss without any detectable interactions with a CAR T cell during the imaging period. For determining the area occupied by tumor cells, by apoptotic tumors and by CAR T cells from in vivo images, each fluorescent channel was binarized and the corresponding signals were quantified by Fiji software.

For STAT1 translocation analysis, a coefficient of variation was calculated as the ratio of the s.d. to the mean of the STAT1–GFP signal for each cell. A translocation index was then defined as 1 / coefficient of variation and reflected the homogeneity of STAT1–GFP distribution in individual tumor cells.

### Patients and immune monitoring

We studied 63 consecutive patients (mean age 62 years; 22 women and 41 men) with aggressive DLBCL who received commercial axicabtagene ciloleucel (axi-cel, *n* = 35) or tisagenlecleucel (tisa-cel, *n* = 28) between April 2019 and September 2020 in Saint-Louis Hospital. One-year estimates of PFS and OS were evaluated based on the Lugano criteria^70^. Parameters of immune monitoring were routinely determined as a standard of care. Peripheral blood expansion of CD4^+^ and CD8^+^ CAR T cells was determined every 2 d during the first month after injection by flow cytometry using CD19 CAR detection reagent (Miltenyi Biotech) as reported^71^. Serum IFN-γ levels (Ella, ProteinSimple) were measured at baseline (day 0) and around the peak of CAR T-cell expansion (day 7). The study was performed under institutional review-board-approved protocols and all patients signed informed consent.

### Statistics and reproducibility

No statistical method was used to predetermine sample size but our sample sizes are similar to those reported in previous publications. The experiments were not randomized. The investigators were not blinded to allocation during experiments and outcome assessment. No data were excluded from the analyses. For two groups statistical testing, data met the assumptions of the statistical tests used. All statistical tests were performed using Prism v.9.2.0 (GraphPad). Data are expressed as mean ± s.e.m. Unpaired Student’s *t*-test, Mann–Whitney *U*-test, one-way ANOVA, two-way ANOVA and log-rank tests were used as indicated in figure legends, using post hoc Tukey and Holm-Sidak test for multiple comparison correction. All statistical tests were two-tailed with a significance level of 0.05. NS, not significant; *P < 0.05; **P < 0.01; ***P < 0.001.

### Reporting summary

Further information on research design is available in the Nature Portfolio Reporting Summary linked to this article.

## Supplementary information

Reporting Summary

Supplementary Video 1 Examples of direct and indirect tumor killing during CAR4 T-cell therapy. C57BL/6 mice were sublethally irradiated and injected with Pro-B-DEVD cell tumors 6 d before treatment with GFP-expressing CAR4 T cells. After 2–3 d, intravital imaging of the bone marrow was performed. The video shows one example of a tumor cell undergoing apoptosis following a direct cellular contact with a CAR4 T cell (direct killing events) and one example of tumor cells undergoing apoptosis without apparent contact with CAR4 T cells (indirect killing events). The white circle highlights the tumor cell undergoing apoptosis following a cellular contact with a CAR4 T cell. The red circles highlight tumor cells undergoing apoptosis without apparent contact with CAR4 T cells. CAR4 T cells are shown in green, live tumor cells in white and apoptotic tumor cells in blue. Scale bars, 10 µm.

Supplementary Video 2 WT CAR4 T cells eliminate tumors using both contact-dependent and independent mechanisms. C57BL/6 mice were sublethally irradiated and injected with Pro-B-DEVD cell tumors 6 d before treatment with GFP-expressing WT CAR4 T cells. After 3 d, intravital imaging of the bone marrow was performed. The white circles highlight tumor cells undergoing apoptosis following a direct cellular contact with a WT CAR4 T cell (direct killing events). The red circles highlight tumor cells undergoing apoptosis without apparent contact with WT CAR4 T cells (indirect killing events). CAR4 T cells are shown in green, live tumor cells in white and apoptotic tumor cells in blue. Scale bars, 30 µm. Total duration 1 h 15 min.

Supplementary Video 3 IFN-γ^−/−^ CAR4 T cells primarily induce direct, contact-dependent tumor killing. C57BL/6 mice were sublethally irradiated and injected with Pro-B-DEVD cell tumors 6 d before treatment with GFP-expressing IFN-γ^−/−^ CAR4 T cells. After 3 d, intravital imaging of the bone marrow was performed. The white circles highlight tumor cells undergoing apoptosis following a direct cellular contact with an IFN-γ^−/−^ CAR4 T cell (direct killing events). The red circle highlights a tumor cell undergoing apoptosis without apparent contact with IFN-γ^−/−^ CAR4 T cells (indirect killing events). CAR4 T cells are shown in green, live tumor cells in white and apoptotic tumor cells in blue. Scale bars, 30 µm. Total duration 1 h 6 min.

Supplementary Video 4 Prf1^−/−^ CAR4 T cells primarily induce indirect tumor cell killing. C57BL/6 mice were sublethally irradiated and injected with Pro-B-DEVD cell tumors 6 d before treatment with GFP-expressing Prf1^−/−^ CAR4 T cells. After 2 d, intravital imaging of the bone marrow was performed. The white circle highlights a tumor cell undergoing apoptosis following a direct cellular contact with a Prf1^−/−^ CAR4 T cell (direct killing events). The red circles highlight tumor cells undergoing apoptosis without apparent contact with Prf1^−/−^ CAR4 T cells (indirect killing events). CAR4 T cells are shown in green, live tumor cells in white and apoptotic tumor cells in blue. Scale bars, 30 µm. Total duration 59 min.

Supplementary Video 5 Indirect tumor killing events are not the result of perforin-dependent elimination by host cytotoxic effectors. Prf1^−/−^ mice were sublethally irradiated and injected with Pro-B-DEVD cell tumors 6 d before treatment with GFP-expressing WT CAR4 T cells. After 2 d, intravital imaging of the bone marrow was performed. The white circle highlights a tumor cell undergoing apoptosis following a direct cellular contact with a WT CAR4 T cell (direct killing events). The red circles highlight tumor cells undergoing apoptosis without apparent contact with WT CAR4 T cells (indirect killing events). CAR4 T cells are shown in green, live tumor cells in white and apoptotic tumor cells in blue. Scale bars, 30 µm. Total duration 59 min.

## Data Availability

Source data have been provided as Source Data files. All other data supporting the findings of the present study are available from the corresponding author on reasonable request. Source data are provided with this paper.

## Source data

Source Data Fig. 1 Statistical Source Data for Fig. 1.
Source Data Fig. 2 Statistical Source Data for Fig. 2.
Source Data Fig. 3 Statistical Source Data for Fig. 3.
Source Data Fig. 4 Statistical Source Data for Fig. 4.
Source Data Fig. 5 Statistical Source Data for Fig. 5.
Source Data Fig. 6 Statistical Source Data for Fig. 6.
Source Data Fig. 7 Statistical Source Data for Fig. 7.
Source Data Extended Data Fig. 1 Statistical Source Data for Extended Data Fig. 1.
Source Data Extended Data Fig. 4 Statistical Source Data for Extended Data Fig. 4.
Source Data Extended Data Fig. 5 Statistical Source Data for Extended Data Fig. 5.
Source Data Extended Data Fig. 6 Statistical Source Data for Extended Data Fig. 6.
Source Data Extended Data Fig. 7 Statistical Source Data for Extended Data Fig. 7.
Source Data Extended Data Fig. 8 Statistical Source Data for Extended Data Fig. 8.
Source Data Extended Data Fig. 9 Statistical Source Data for Extended Data Fig. 9.
Source Data Extended Data Fig./ 10 Statistical Source Data for Extended Data Fig. 10.
