# Supplementary material for: Tumor-intrinsic sensitivity to the pro-apoptotic effects of IFN-γ is a major determinant of CD4+ CAR T-cell antitumor activity
Source: Nat Cancer. 2023 May 29;4(7):968–83. doi: 10.1038/s43018-023-00570-7 (PMC10368531; doi:10.1038/s43018-023-00570-7)
Supplement: Supplementary file 1 — Reporting Summary [file 43018_2023_570_MOESM1_ESM.pdf]

nature portfolio

Corresponding author(s): Philippe Bousso

Last updated by author(s): Apr 2, 2023

Reporting Summary

Nature Portfolio wishes to improve the reproducibility of the work that we publish. This form provides structure for consistency and transparency in reporting. For further information on Nature Portfolio policies, see our Editorial Policies and the Editorial Policy Checklist.

Statistics

For all statistical analyses, confirm that the following items are present in the figure legend, table legend, main text, or Methods section.

n/a	Confirmed	
☐	☒	The exact sample size (*n*) for each experimental group/condition, given as a discrete number and unit of measurement
☐	☒	A statement on whether measurements were taken from distinct samples or whether the same sample was measured repeatedly
☐	☒	The statistical test(s) used AND whether they are one- or two-sided<br>*Only common tests should be described solely by name; describe more complex techniques in the Methods section.*
☒	☐	A description of all covariates tested
☒	☐	A description of any assumptions or corrections, such as tests of normality and adjustment for multiple comparisons
☐	☒	A full description of the statistical parameters including central tendency (e.g. means) or other basic estimates (e.g. regression coefficient) AND variation (e.g. standard deviation) or associated estimates of uncertainty (e.g. confidence intervals)
☐	☒	For null hypothesis testing, the test statistic (e.g. *F*, *t*, *r*) with confidence intervals, effect sizes, degrees of freedom and *P* value noted<br>*Give P values as exact values whenever suitable.*
☒	☐	For Bayesian analysis, information on the choice of priors and Markov chain Monte Carlo settings
☒	☐	For hierarchical and complex designs, identification of the appropriate level for tests and full reporting of outcomes
☒	☐	Estimates of effect sizes (e.g. Cohen's *d*, Pearson's *r*), indicating how they were calculated

Our web collection on statistics for biologists contains articles on many of the points above.

Software and code

Policy information about availability of computer code

Data collection	For FACS experiments, samples were acquired using CytExpert 2.3 software (Beckman Coulter).<br>For microscopy experiments, movies were acquired using FV31S-SW software (Olympus v2.3.1.163).
Data analysis	Flow cytometry data were analyzed using FlowJo v10.8.1 (Tree Star).<br>Microscopy movies were created from raw data and analyzed using Fiji software (Image J v2.3.0).<br>Data were represented using GraphPad Prism v9.2.0.

For manuscripts utilizing custom algorithms or software that are central to the research but not yet described in published literature, software must be made available to editors and reviewers. We strongly encourage code deposition in a community repository (e.g. GitHub). See the Nature Portfolio guidelines for submitting code & software for further information.

Data

Policy information about availability of data

All manuscripts must include a data availability statement. This statement should provide the following information, where applicable:

- Accession codes, unique identifiers, or web links for publicly available datasets
- A description of any restrictions on data availability
- For clinical datasets or third party data, please ensure that the statement adheres to our policy

Source data have been provided as Source Data files. All other data supporting the findings of the present study are available from the corresponding author on reasonable request.

Human research participants

Policy information about studies involving human research participants and Sex and Gender in Research.

Reporting on sex and gender	Sex was not considered in the study design. Gender was not considered in the study design (gender is not a criterium in the decision to treat with CAR T cells).
Population characteristics	We studied 63 consecutive patients (mean age 62 years, 22 women/41 males) with aggressive diffuse large B cell lymphoma (DLBCL) who received commercial CAR T cells between April 2019 and September 2020 in Saint-Louis Hospital (Paris, France).
Recruitment	Patients with aggressive DLBCL were recruited at Saint-Louis Hospital (Paris, France) between April 2019 and September 2020. The study was performed under institutional review board (IRB)-approved protocols, and all patients signed informed consent.
Ethics oversight	BIOCART-CPP 2019-77, IRB St-Louis hospital

Note that full information on the approval of the study protocol must also be provided in the manuscript.

Field-specific reporting

Please select the one below that is the best fit for your research. If you are not sure, read the appropriate sections before making your selection.

☒ Life sciences ☐ Behavioural & social sciences ☐ Ecological, evolutionary & environmental sciences

For a reference copy of the document with all sections, see nature.com/documents/nr-reporting-summary-flat.pdf

Life sciences study design

All studies must disclose on these points even when the disclosure is negative.

Sample size	No statistical method was used to predetermine sample size but our sample sizes are similar to those reported in previous publications.
Data exclusions	No data were excluded from the analysis.
Replication	Experiments were repeated several times, as indicated in figure legends. All repetitions of the experiments yielded similar results. Mice were housed in the same facility and were age-matched as best as possible in every experiment and between different experiments. At the time of injection, tumor cells were less than 6 weeks in culture.
Randomization	Age- and sex-matched mice were used. The experiments were not randomized.
Blinding	The experimentators were not blinded to experimental conditions during experiments or analysis. Experiments and analyses were often performed by the same person.

Reporting for specific materials, systems and methods

We require information from authors about some types of materials, experimental systems and methods used in many studies. Here, indicate whether each material, system or method listed is relevant to your study. If you are not sure if a list item applies to your research, read the appropriate section before selecting a response.

Materials & experimental systems

n/a	Involved in the study	
☐	☒	Antibodies
☐	☒	Eukaryotic cell lines
☒	☐	Palaeontology and archaeology
☐	☒	Animals and other organisms
☒	☐	Clinical data
☒	☐	Dual use research of concern

Methods

n/a	Involved in the study	
☒	☐	ChIP-seq
☐	☒	Flow cytometry
☒	☐	MRI-based neuroimaging

Antibodies

Antibodies used

The following antibodies were used:
- anti-mouse CD3 (clone 17.A2; BioLegend, #100201, 2.5 µg/mL)
- anti-mouse CD28 (clone 37.51; BioLegend, #102101, 2.5 µg/mL)
- anti-mouse CD16/32 (clone 93; BioLegend, #101301, 1:100 dilution)
- anti-human CD34-PE (clone 561; BioLegend, #343605, 1:25 dilution)
- anti-human CD34-Alexa Fluor 647 (clone 561; BioLegend, #343617, 1:25 dilution)
- anti-mouse CD4-BUV395 (clone GK1.5; BD Biosciences, #565974, 1:100 dilution)
- anti-mouse CD4-BV786 (clone RM4-5; BD Biosciences, #563727, 1:100 dilution)
- anti-mouse CD8a-BUV395 (clone 53-6.7; BD Biosciences, #563786, 1:100 dilution)
- anti-mouse CD11b-BUV395 (clone M1/70; BD Biosciences, #565976, 1:100 dilution)
- anti-mouse CD19-PE/Cy7 (clone 6D5; BioLegend, #115519, 1:100 dilution)
- anti-mouse CD19-APC-fire750 (clone 6D5; BioLegend, #115557, 1:100 dilution)
- anti-mouse CD44-Alexa488 (clone IM7; BioLegend, #103015, 1:100 dilution)
- anti-mouse CD45.1-PE (clone A20; BioLegend, #110707, 1:100 dilution)
- anti-mouse CD45.2-BUV737 (clone 104; BD Biosciences, #612779, 1:100 dilution)
- anti-mouse CD62L-BV421 (clone MEL-14; BioLegend, #104435, 1:100 dilution)
- anti-mouse H-2Db-APC (clone KH95; BioLegend, #111513, 1:100 dilution)
- anti-mouse H-2Kb-PE/Cy7 (clone AF6-88.5; BioLegend, #116519, 1:100 dilution)
- anti-mouse LAG-3-APC-fire750 (clone C9B7W; BioLegend, #125239, 1:100 dilution)
- anti-mouse PD-1-PE/Cy7 (clone J43; eBiosciences, #25-9985-82, 1:100 dilution)
- anti-mouse PD-L1-APC (clone 10F.9G2; BioLegend, #124311, 1:100 dilution)
- anti-mouse PD-L1-BV785 (clone 10F.9G2; BioLegend, #124331, 1:100 dilution)
- anti-mouse TIGIT-PE (clone GIGD7; eBiosciences, #12-9501-82, 1:100 dilution)
- anti-mouse Tim-3-BV785 (clone RMT3-23; BioLegend, #119725, 1:100 dilution)
- anti-mouse CD54 (ICAM-1)-biotin (clone 3E2; BD Biosciences, #553251, 1:100 dilution)
- anti-mouse CD119 (IFN-g-R1)-biotin (clone 2E2; BioLegend, #112803, 1:100 dilution)
- streptavidin-PE (BioLegend, #405203, 1:200 dilution)
- streptavidin-APC-fire750 (BioLegend, #405250, 1:200 dilution)
- anti-human/mouse Active caspase 3-Alexa Fluor 647 (clone C92-605; BD Biosciences, #560626, 1:50 dilution)
- anti-human CD19-APC (clone HIB19; BioLegend, #302211, 1:50 dilution)
- anti-human HLA-A,B,C-BV605 (clone W6/32; BioLegend, #311431, 1:100 dilution)
- anti-human PD-L1-PE (clone 29E.2A3; BioLegend, #329705, 1:25 dilution)

Antibodies used in vitro for blocking purposes:
- anti-mouse IFN-g (clone XMG1.2; BioLegend, #505801)
- anti-mouse CD253 (TRAIL) (clone N2B2; eBioscience, #15297617)
- anti-mouse/rat CD178 (FasL) (clone MFL4; BioLegend, #106707)
- anti-human IFN-g (clone NIB42; BD Biosciences, #551221)
- anti-human IFN-g-R1 (clone 92101; R&D Systems, #MAB6731)

Validation

All antibodies were validated by the corresponding manufacturers for that specific application (flow cytometry). For each experiment, unstained controls were added to validate the positivity of the stainings. Relevant information on antibodies validation can be found on the manufacturer's website for each antibody. Most primary antibodies have further been validated on lymph node cells.

Eukaryotic cell lines

Policy information about cell lines and Sex and Gender in Research

Cell line source(s)

The Eµ-myc cell line was derived in our laboratory from a male Eµ-myc transgenic mouse. Eµ-myc mice harbor a transgene with the c-myc gene under the IgH promoter and develop spontaneous B lymphoma. Immortalized Pro-B cells lines were generated by infecting bone marrow cells (from different male mice) with a retrovirus encoding the viral-Abelson kinase. HEK cells were purchased at American Type Culture Collection. B16.F10 melanoma cells, purchased at American Type Culture Collection, are a gift from Guy Shakhar (Weizmann Institute, Rehovot, Israel). MC38 cells were purchased at Kerafast. OVCAR3 ovarian adenocarcinoma cells, purchased at American Type Culture Collection, are a gift from Eliane Piaggio (Institut Curie, France).

Authentication

HEK, B16.F10 and OVCAR3 cells were previously authenticated by ATCC (see vendor website). The MC38 cells were previously

Authentication	authenticated by Kerafast (see vendor website). The phenotype of the cell lines used in the study was checked after thawing of the cells based on their morphology and phenotypic analyses by flow cytometry.
Mycoplasma contamination	All cell lines were routinely tested negative for mycoplasma contamination by PCR.
Commonly misidentified lines (See ICLAC register)	No commonly misidentified cell lines were used.

Animals and other research organisms

Policy information about studies involving animals; ARRIVE guidelines recommended for reporting animal research, and Sex and Gender in Research

Laboratory animals	C57BL/6 (B6) mice were obtained from Envigo. Ifng-deficient (B6.129S7-Ifngtm1Ts/J), IfngR1-deficient (B6.129S7-Ifngr1tm1Agt/J), Prf1deficient (C57BL/6-Prf1tm1Sdz/J), ROSAmT/mG (Gt(ROSA)26Sortm4(ACTB-tdTomato -EGFP)Luo/J), Ubi-GFP (C57BL/6-Tg(UBC-GFP)30Scha/J), CFP (Tg(ACTB-ECFP)CK6Nagy* (Tg/Tg)) and CD45.1 (B6.SJL-Ptprca Pepcb/BoyJ) mice were bred and crossed in our animal facility under specific pathogen-free conditions<br>Only age-matched mice were used.<br>Mice were 6 to 8-week-old at the time of the experiments and all experiments were performed in agreement with relevant Institut Pasteur internal guidelines and regulations (CETEA comittee, protocol number 170038).
Wild animals	The study did not involve wild animals
Reporting on sex	Age-matched mice were used. Male mice were used when using male tumor cell lines. Female mice were used when using female tumor cell lines.
Field-collected samples	The study did not involved field-collected samples.
Ethics oversight	All experiments were carried out in agreement with relevant guidelines and regulations and approved by the Institut Pasteur committee on Animal Welfare (CETEA) under the protocol code of CETEA 2017-0038.

Note that full information on the approval of the study protocol must also be provided in the manuscript.

Flow Cytometry

Plots

Confirm that:

☒ The axis labels state the marker and fluorochrome used (e.g. CD4-FITC).

☒ The axis scales are clearly visible. Include numbers along axes only for bottom left plot of group (a 'group' is an analysis of identical markers).

☒ All plots are contour plots with outliers or pseudocolor plots.

☒ A numerical value for number of cells or percentage (with statistics) is provided.

Methodology

Sample preparation	For ex vivo analysis, bone marrow cells were isolated by flushing femurs and tibias from tumor-bearing mice and subsequent filtering through 70 µm cell strainers. Single-cell suspensions from spleen were prepared by filtering the cells through 70 µm cell strainers. Blood was collected by cardiac puncture after mouse sacrifice and red blood cells were removed using red blood cell lysis buffer (eBiosciences). When indicated, cells were stained with live/dead (Fixable Viability Dye eFluor 780 (eBioscience) or Zombie NIR Fixable Viability Kit (BioLegend)) staining during 20 min at room temperature. Then single-cell suspensions were Fc-blocked using anti-CD16/32 mAbs (clone 93; BioLegend) and normal murine serum 1%.
Instrument	Data were acquired using a Cytoflex LX (serial number BA42021, Beckman Coulter).
Software	CytExpert version 2.3 was used on Cytoflex cytometer.
Cell population abundance	No cell sorting was performed during experiments.
Gating strategy	For experiments using Eµ-myc B cell lymphoma or Pro-B cell tumors in vivo, cell doblets were gated out based on SSC-A/SSC-H followed by an additional gating based on FSC-A/FSC-H. Live cells were isolated by FSC-A/SSC-A gating or based on a live/dead staining. CFP and GFP were used to detect tumor cells expressing the FRET-based probe for caspase-3 activity. Tumor apoptosis was quantified using a FRET-based reporter for caspase 3 activity, and FRET loss was defined as a derived parameter using the ratio of CFP to FRET fluorescence. FRET loss was measured on cells fixed with 2% paraformaldehyde solution (Sigma) immediately after ex vivo isolation. Pro-B cell tumors deficient for IFN-g-R1 were identified based on their CD45.2 expression. For Pro-B cell tumors expressing GFP or YFP or CFP or mTom, their respective fluorescence were used for identification.

☒ Tick this box to confirm that a figure exemplifying the gating strategy is provided in the Supplementary Information.
